# Efficient gene editing in *Neurospora crassa* with CRISPR technology

**DOI:** 10.1186/s40694-015-0015-1

**Published:** 2015-07-15

**Authors:** Toru Matsu-ura, Mokryun Baek, Jungin Kwon, Christian Hong

**Affiliations:** 1 Department of Molecular and Cellular Physiology, University of Cincinnati, 231 Albert Sabin Way, Cincinnati, OH 45267-0576 USA; 2 Division of Developmental Biology, Cincinnati Children’s Hospital Medical Center, 3333 Burnet Avenue, Cincinnati, OH 45229-3026 USA

**Keywords:** *Neurospora crassa*, CRISPR/Cas9, Genome editing, Homologous recombination, Biofuel, Cellulase

## Abstract

**Background:**

Efficient gene editing is a critical tool for investigating molecular mechanisms of cellular processes and engineering organisms for numerous purposes ranging from biotechnology to medicine. Recently developed RNA-guided CRISPR/Cas9 technology has been used for efficient gene editing in various organisms, but has not been tested in a model filamentous fungus, *Neurospora crassa*.

**Findings:**

In this report, we demonstrate efficient gene replacement in a model filamentous fungus, *Neurospora crassa*, with the CRISPR/Cas9 system. We utilize Cas9 endonuclease and single crRNA:tracrRNA chimeric guide RNA (gRNA) to: (1) replace the endogenous promoter of *clr*-*2* with the *β*-*tubulin* promoter, and (2) introduce a codon optimized fire fly *luciferase* under the control of the *gsy*-*1* promoter at the *csr*-*1* locus. CLR-2 is one of the core transcription factors that regulate the expression of cellulases, and GSY-1 regulates the conversion of glucose into glycogen. We show that the *β*-*tubulin* promoter driven *clr*-*2* strain shows increased expression of cellulases, and *gsy*-*1*-*luciferase* reporter strain can be easily screened with a bioluminescence assay.

**Conclusion:**

CRISPR/Cas9 system works efficiently in *Neurospora crassa*, which may be adapted to Neurospora natural isolates and other filamentous fungi. It will be beneficial for the filamentous fungal research community to take advantage of CRISPR/Cas9 tool kits that enable genetic perturbations including gene replacement and insertions.

**Electronic supplementary material:**

The online version of this article (doi:10.1186/s40694-015-0015-1) contains supplementary material, which is available to authorized users.

## Findings

Genetic engineering of organisms of interest are critical tools to elucidate molecular and cellular processes or to engineer organisms with new characteristics or traits. Clustered regularly interspaced short palindromic repeats (CRISPR)-associated RNA-guided DNA endonuclease, Cas9, has been driving the latest gene editing technology by taking advantage of the simple design of a single crRNA:tracrRNA chimeric guide RNA (gRNA). gRNA includes 20 base pairs of target sequences that causes double strand breaks by Cas9 at the target locus, which triggers gene replacement by homologous recombination (HR) [1–3] (reviewed by Hsu et al. [4]). CRISPR/Cas9 system has been utilized for gene editing ranging from yeast to human cells including filamentous fungi [5–8].

A model filamentous fungus, *Neurospora crassa*, has been used to elucidate fundamental molecular mechanisms including the one gene-one enzyme hypothesis, cell fusion, circadian rhythms, and epigenetics among others [9]. Gene editing by HR was a difficult task in *N. crassa* until the discovery of highly efficient gene replacement in Neurospora strains lacking *mus*-*51* (NCU08290) or *mus*-*52* (NCU00077), which are deficient in non-homologous end-joining [10]. This finding facilitated the high-throughput generation of single gene deletion knockout (KO) collection of *N. crassa* [11]. However, one has to backcross Neurospora transformants to wild type in order to eliminate *mus*-*51* or *mus*-*52* KO background, which may take anywhere between one to three months. More importantly, there are numerous Neurospora natural isolates that are difficult to generate initial *mus*-*51* or *mus*-*52* KO, which make gene editing difficult. Therefore, it is critical to establish an alternative gene editing technology for the Neurospora system.

**Figure 1:**
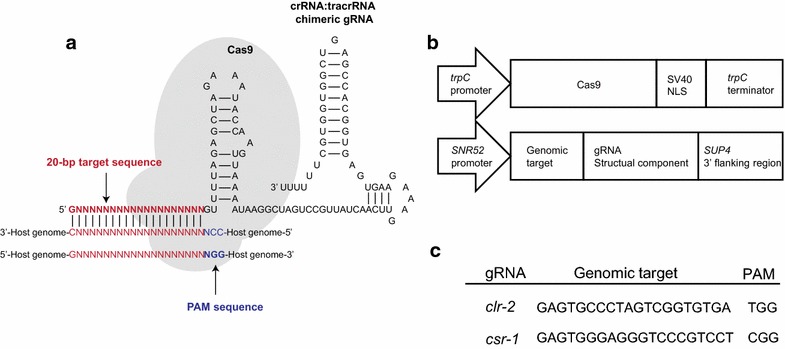
System overview of genomic edition in *N. crassa* using CRISPR/Cas9. **a** The system consists of two components, a Cas9 protein and a single crRNA:tracrRNA chimeric guide RNA (gRNA), comprising a 20-bp target sequence (*red*) complementary to the genomic target adjacent to a PAM site of NGG (*blue*). **b** Design of the Cas9 and gRNA constructs. The Cas9 protein contained a SV40 nuclear localization signal, and the expression was under the control of the *trpC* promoter and terminator. The gRNA was expressed under the snoRNA *SNR52* promoter and contained a terminator from the 30 region of the yeast *SUP4* gene. **c** Design of gRNA targeted to *clr*-*2* and *csr*-*1* loci.

The design of the CRISPR/Cas9 system consists of Cas9 DNA endonuclease and a guide RNA (gRNA) (Figure 1a). Cas9 is fused to the *trpC* (AN0648) promoter from the *Aspergillus nidulans* followed by both SV40 nuclear localization signal (NLS) domain and the *trpC* terminator sequence from the *A. nidulans* (Figure 1b) [12]. gRNA is fused to Small Nucleolar RNA 52 (*SNR52*) promoter from the *Saccharomyces cerevisiae* [13] and includes specific 20 base pair target sequences followed by gRNA structural component and *SUP4* flanking region (Figure 1b). The promoter of *SNR52* has been used to express gRNA in *S. cerevisiae* [14]. We designed target sequences for *clr*-*2* (NCU08042) and *csr*-*1* (NCU00726) loci followed by protospacer adjacent motif (PAM) (Figure 1c), which is required for target recognition.

**Figure 2:**
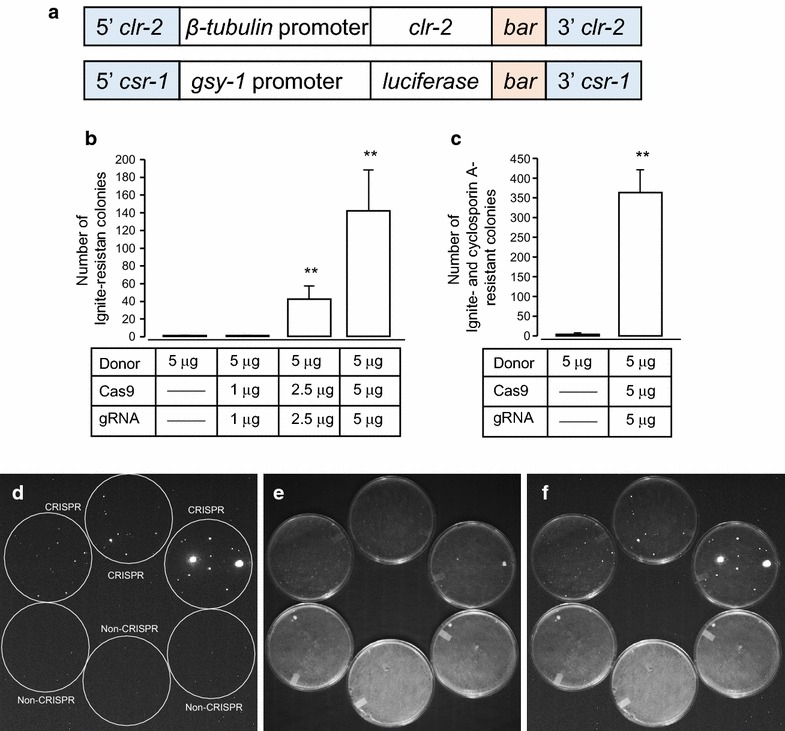
Evaluation of CRISPR/Cas9 system for transformation of *N. crassa*. **a** Diagram of donor plasmids. Blue color regions are the sequence regions that are homologous to genomic DNA. *Bar* gene cassette which contains the *bar* gene under the control of *trpC* promoter and terminator was included in each plasmids for selection. **b** The number of Ignite-resistant colonies by *clr*-*2* locus targeted transformation with different amount of Cas9 and gRNA plasmids. From left to right: zero, 1, 2.5, and 5 µg each of Cas9 and gRNA plasmid was co-transfected with the donor plasmid (5 µg). **p < 0.01, Tukey’s test. *Error bars* corresponds to the SEM. **c** The number of Ignite-resistant colonies by *csr*-*1* locus targeted transformation with zero and 5 µg each of Cas9 and gRNA plasmids. **p < 0.01, student’s t-test. *Error bars* corresponds to the SEM. **d**–**f** Luciferase activity on the plates of *gsy*-*1*-*luciferase* transformants. Luciferase signal (**d**), under red-light (**e**), and merged (**f**) images are shown.

Cas9 and gRNA were constructed using PCR and yeast recombination (Additional file 1: Figure S1), and both Cas9 and gRNA were transiently transfected with a donor vector/plasmid. Donor plasmids were constructed using PCR and yeast recombination (Additional file 1: Figure S1, Figure 2a). We designed two separate constructs to: (1) drive the expression of *clr*-*2* with *β*-*tubulin* (NCU04054) promoter, and (2) drive the expression of codon optimized fire fly *luciferase* [15] with the *glycogen synthase*-*1* (*gsy*-*1*: NCU06687) promoter. CLR-2 is a zinc binuclear cluster transcription factor that regulates a large number of cellulases in *N. crassa* [16], and Glycogen Synthase-1 (GSY-1) is an enzyme that converts glucose into glycogen [17]. *β*-*tubulin*-*clr*-*2* and *gsy*-*1*-*luciferase* sequences were followed by the *bar* gene for selection using Ignite^®^ (glufosinate), and they were targeted to its endogenous locus and *csr*-*1* locus, respectively. *csr*-*1* is a commonly used locus for transformation in *N. crassa*. Gene replacement of the *csr*-*1* gene, which encodes the cyclosporin A-binding protein, leads to resistance to cyclosporin A [18]. The strains used in this study are listed in Table 1.

**Table 1:** Strains used in this study

Strain	FGSC# or reference	Mating type	Genotype
74-OR23-1V A	987	A	Wild type (WT)
328-4	Belden et al. [20]	a	*ras*-*1* bd
*mus*-*51* KO	20277	a	Δ*mus*-*51*

As a proof of principle, we set out to test overexpression of cellulases with the *β*-*tubulin* promoter-driven expression of *clr*-*2,* and efficient selection of luciferase reporters with a bioluminescence assay. 5 μg of *β*-*tubulin*-*clr*-*2* circular donor plasmids were transformed into wild type *N. crassa* (74-OR23-1 V A) along with different concentrations of Cas9 and gRNA circular plasmids (Figure 2b). Transformations were performed as previously described [19]. Dose-dependent increase of the number of transformants of *β*-*tubulin*-*clr*-*2* was obtained by addition of Cas9 and gRNA (Figure 2b; Cas9 and gRNA plasmids: 0 µg: 1.0 ± 0.6; 1 µg: 0.8 ± 0.5; 2.5 µg: 42.0 ± 29.7; 5 µg 141.8 ± 92.9; n = 3, p < 0.01; Tukey’s method). We utilized optimal concentrations (5 µg) of Cas9 and gRNA with *gsy*-*1*-*luciferase::csr*-*1* donor plasmid to transform the *ras*-*1* ^*bd*^ *N. crassa* strain [20] (Figure 2c). *gsy*-*1*-*luciferase::csr*-*1* that targets *csr*-*1* locus enables double selection for both Ignite^®^ and cyclosporine A as previously described [21]. As expected, we observed an efficient number of transformants of *gsy*-*1*-*luciferase::csr*-*1* with optimized concentrations (Figure 2c; Cas9 and gRNA plasmids: 0 µg: 3.7 ± 1.9; 1 µg: 364.0 ± 33.1; n = 3, p < 0.01; Student’s *t* test). The transformants produced with the CRISPR/Cas9 system successfully express the transgene. The transformed conidia were plated onto luciferin containing plates, and in vivo luciferase activities were monitored with a charge-coupled device camera. We observed 57 luciferase positive colonies out of 1,092 total colonies with CRISPR/Cas9 system, but no positive colonies were observed with non-CRISPR/Cas9 method of transformation [11, 19] (Figure 2d–f). The reason for low number of positive colonies with bioluminescence assay is due to the small sizes of other colonies, which do not produce detectable signals.

**Figure 3:**
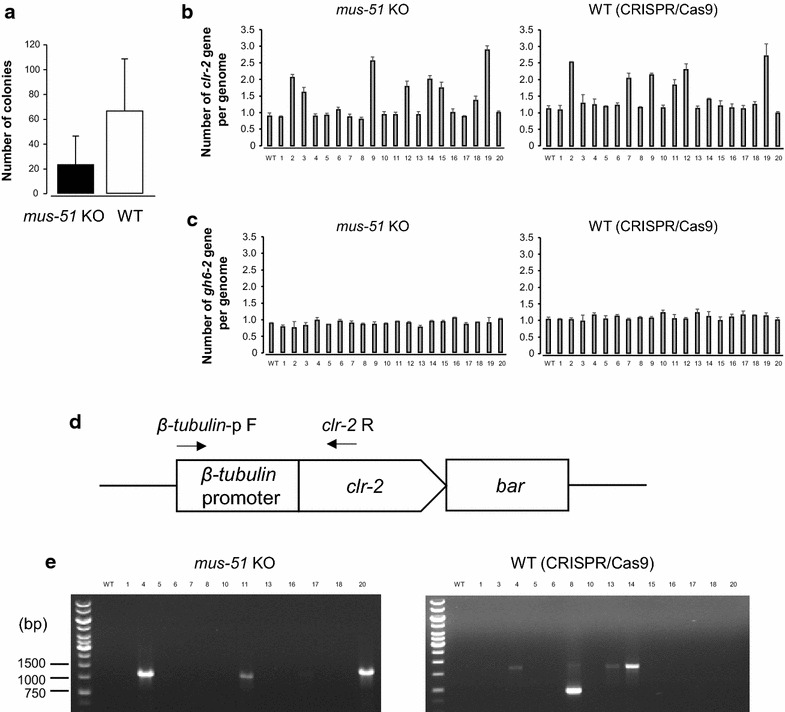
Rate of homologous integration at *clr*-*2* locus in wild type with CRISPR/Cas9 technology versus *mus*-*51* KO with the traditional method. **a** The number of Ignite-resistant colonies by *clr*-*2* locus targeted transformation in the *mus*-*51* KO and wild type (WT: 74-OR23-1V A) with CRISPR/Cas9. *Error bars* corresponds to the SEM. **b** qPCR analysis to assess the number of *clr*-*2* in genomic DNA from the transformants with either WT (*right panel*) or *mus*-*51* KO backgrounds (*left panel*). *Error bars* corresponds to the SEM. **c** qPCR analysis to assess the number of *gh6*-*2* in genomic DNA from the transformants with either WT (*right panel*) or *mus*-*51* KO backgrounds (*left panel*). *Error bars *corresponds to the SEM. **d** Schematic overview of the priming sites for PCR analysis to confirm the connection of *β*-tubulin promoter and *clr*-*2*. **e** PCR assay using *β*-*tubulin*-p F and *clr*-*2* R primers. Expected fragment size: 1,343 bp.

Next, we assessed the efficiency of HR in 74-OR23-1V A with the CRISPR/Cas9 system versus *mus*-*51* KO with the traditional method [10]. We observed efficient transformations with both methodologies (Figure 3a; *mus*-*51* KO: 23 ± 56; CRISPR/Cas9: 66 ± 103; n = 4). Successful transformants at the *clr*-*2* locus were validated through polymerase-chain reaction (PCR) analysis (see Table 2 for primers used in this study). The transformants were grown in liquid culture media, and we did not observe any differences of growth between the transformants from both methodologies. The genomic DNA was extracted from the transformants with either 74-OR23-1V A or *mus*-*51* KO background, and quantitative PCR (qPCR) analysis was performed to count the number of transgenes in the genome. Similar number of transformants showed more than one *clr*-*2* in their genome from both methods (CRISPR/Cas9: 6/20; *mus*-*51* KO: 7/20; Figure 3b). As a control, we tested the number of *gh6*-*2* in each genome and confirmed that none of the transformants had more than one *gh6*-*2* (Figure 3c). The transformants with single *clr*-*2* in their genome were further validated by PCR to confirm the *β*-*tubulin* promoter fused to *clr*-*2* gene (Figure 3d). Interestingly, we observed three transformants from each method that exhibit the correct HR events at the *clr*-*2* locus (CRISPR/Cas9: 3/14; *mus*-*51* KO: 3/13; Figure 3e).

**Table 2:** Primers used in this study

Primer name	Sequence 5′-3′	Used for
*β*-*tubulin*-p F	TGCGACCAGGTTCAGGAGAGC	Genomic PCR (Figure 3e)
*actin* F	GTCCCCGTCATCATGGTATC	qRT-PCR (Figure 4)
*actin* R	CTTCTCCATGTCGTCCCAGT	qRT-PCR (Figure 4)
*clr*-*2* F	GCACCATCAATGTCGATACCTAC	qPCR, qRT-PCR (Figures 3b; 4a)
*clr*-*2* R1	CATTGGCCACATGGTTGTTGACC	qPCR (Figure 3b, e)
*clr*-*2* R2	CCATCACACCGAATCTTTCGTCT	qRT-PCR (Figure 4a)
*cbh*-*1* F	CTGCGTTGATGGTGCTGAGTAC	qRT-PCR (Figure 4b)
*cbh*-*1* R	GAGCTCGAAGCCCTGGTAGG	qRT-PCR (Figure 4b)
*gh5*-*1* F	CTCCTGCTAGCACCACCACTG	qPCR, qRT-PCR (Figures 3b, c; 4c)
*gh5*-*1* R1	CAAGGCGCTCCATGGCGAAG	qPCR (Figure 3b, c)
*gh5*-*1* R2	CTGCGGAGAGTCTGGATGGTG	qRT-PCR (Figure 4c)
*gh6*-*2* F	GCTCTGCCTGGAGCCAGTG	qPCR, qRT-PCR (Figures 3c; 4d)
*gh6*-*2* R1	GTGGTGGTAACCTGAGCGCC	qPCR (Figure 3c)
*gh6*-*2* R2	CTTGCTGGTGGTGCTAGAG	qRT-PCR (Figure 4d)

**Figure 4:**
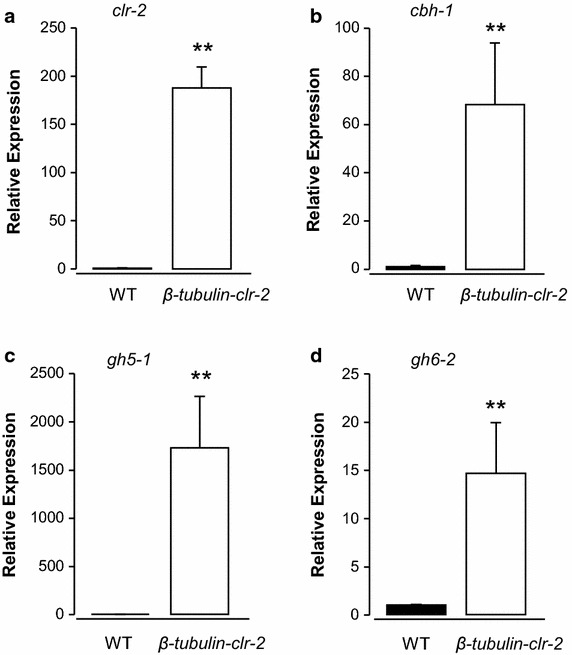
Amplified expression of cellulase genes by enhanced expression of *clr*-*2* with *β*-*tubulin* promoter. The mRNA expression of *clr*-*2* and cellulase genes are measured by qRT-PCR. The expression of *clr*-*2* (**a**), *cbh*-*1* (**b**), *gh5*-*1* (**c**), and *gh6*-*2* (**d**) are shown. *White* and *black bars* show mRNA expressions in wild type (WT: 74-OR23-1V A) and *β*-*tubulin*-*clr*-*2* strains, respectively. Each strain is cultured in the media containing 2% glucose as a sole carbon source. All the expressions were normalized by the expressions in WT. **p < 0.01, student’s t-test. *Error bars* corresponds to the SEM.

Lastly, we tested the expression of cellulases in *N. crassa* carrying a targeted insertion of the *β*-*tubulin* promoter-regulated *clr*-*2* (transformant #14 from the right panel of Figure 3e). We observed approximately two hundred-fold increase of *clr*-*2* mRNA expression in the *β*-*tubulin*-*clr*-*2* strain compared to wild type in 2% glucose as a sole carbon source, which indicates successful overexpression of *clr*-*2* using the *β*-*tubulin* promoter (Figure 4a; 188.2 ± 52.8-fold; n = 3, p < 0.01; Student’s t-test). Increased expression was found for *cbh*-*1* (NCU07340), *gh5*-*1* (NCU00762), and *gh6*-*2* (NCU09680) mRNA (Figure 4b–d; *cbh*-*1*: 68.3 ± 25.5-fold; *gh5*-*1*: 1724.3 ± 538.1-fold; *gh6*-*2*: 14.6 ± 5.3-fold; n = 3, p < 0.01; Student’s t-test; see Table 2 for primers used in this study). CBH-1 and GH6-2 are exoglucanases, and GH5-1 is an endoglucanase that facilitate degradation of cellulose [22].

In this report, we successfully demonstrated efficient gene replacements utilizing CRISPR/Cas9 technology in a model filamentous fungus, *N. crassa*. *N. crassa* did not indicate any problems in expressing functional Cas9 endonuclease under the *A. nidulans* *trpC* promoter, and the *SNR52* promoter is operational to express gRNA in *N. crassa* as in *S. cerevisiae* [14]. These events enabled us to perform efficient knock-in of *clr*-*2* with *β*-*tubulin* promoter and target *gsy*-*1*-*luciferase* bioluminescence reporter at the *csr*-*1* locus with relatively simple modifications of template plasmids utilizing PCR and yeast recombination. In contrast to previously established gene editing protocols in Neurospora, CRISPR/Cas9 technology did not require *mus*-*51* or *mus*-*52* mutant backgrounds for efficient HR. This technology will enable efficient gene editing of any Neurospora natural isolates. Recent advancement and ramifications of CRISPR/Cas9 technology created numerous resources including multiplex gene replacements, transcriptional control, and inducible gene regulation [4]. Implementation of this technology in fungal organisms will be highly beneficial for the fungal research community.

## Materials and methods

Materials and methods and any associated references are available in the online version of the paper at http://www.fungalbiolbiotech.com/ (Additional file 2).

## Additional files

Additional file1: **Figure S1.** Full plasmid sequences of the Cas9, gRNA, and donor vectors. Green, orange, blue, red, light blue, gray letters indicate the promoter, coding sequence, terminator, gRNA, homologous region, and bar gene cassette, respectively. 20-bp target sequences in gRNA are underlined.

Additional file 2. Materials and methods.

## Abbreviations

CRISPR: clustered regularly interspaced short palindromic repeats
gRNA: single crRNA:tracrRNA chimeric guide RNA
KO: knockout
NLS: nuclear localization signal
*SNR52*: small nucleolar RNA 52
PAM: protospacer adjacent motif
*gsy*-*1*: *glycogen synthase*-*1*
HR: homologous recombination
PCR: polymerase chain reaction
qPCR: quantitative polymerase chain reaction
qRT-PCR: quantitative reverse transcription polymerase chain reaction
SEM: standard error of the mean
n: number of experiments
