# Supplementary material for: Efficient gene editing in Neurospora crassa with CRISPR technology
Source: Fungal Biol Biotechnol. 2015 Jul 15;2:4. doi: 10.1186/s40694-015-0015-1 (PMC5611662; doi:10.1186/s40694-015-0015-1)
Supplement: Supplementary file 1 — Figure S1. Full plasmid sequences of the Cas9, gRNA, and donor vectors. Green, orange, blue, red, light blue, gray letters indicate the promoter, coding sequence, terminator, gRNA, homologous region, and bar gene cassette, respectively. 20-bp target sequences in gRNA are underlined. [file 40694_2015_15_MOESM1_ESM.docx]

***Supplementary Figure***

> p415-PtrpC-Cas9-TtrpC-CYC1t

GACGAAAGGGCCTCGTGATACGCCTATTTTTATAGGTTAATGTCATGATAATAATGGTTTCTTAGGACGGATCGCTTGCCTGTAACTTACACGCGCCTCGTATCTTTTAATGATGGAATAATTTGGGAATTTACTCTGTGTTTATTTATTTTTATGTTTTGTATTTGGATTTTAGAAAGTAAATAAAGAAGGTAGAAGAGTTACGGAATGAAGAAAAAAAAATAAACAAAGGTTTAAAAAATTTCAACAAAAAGCGTACTTTACATATATATTTATTAGACAAGAAAAGCAGATTAAATAGATATACATTCGATTAACGATAAGTAAAATGTAAAATCACAGGATTTTCGTGTGTGGTCTTCTACACAGACAAGATGAAACAATTCGGCATTAATACCTGAGAGCAGGAAGAGCAAGATAAAAGGTAGTATTTGTTGGCGATCCCCCTAGAGTCTTTTACATCTTCGGAAAACAAAAACTATTTTTTCTTTAATTTCTTTTTTTACTTTCTATTTTTAATTTATATATTTATATTAAAAAATTTAAATTATAATTATTTTTATAGCACGTGATGAAAAGGACCCAGGTGGCACTTTTCGGGGAAATGTGCGCGGAACCCCTATTTGTTTATTTTTCTAAATACATTCAAATATGTATCCGCTCATGAGACAATAACCCTGATAAATGCTTCAATAATATTGAAAAAGGAAGAGTATGAGTATTCAACATTTCCGTGTCGCCCTTATTCCCTTTTTTGCGGCATTTTGCCTTCCTGTTTTTGCTCACCCAGAAACGCTGGTGAAAGTAAAAGATGCTGAAGATCAGTTGGGTGCACGAGTGGGTTACATCGAACTGGATCTCAACAGCGGTAAGATCCTTGAGAGTTTTCGCCCCGAAGAACGTTTTCCAATGATGAGCACTTTTAAAGTTCTGCTATGTGGCGCGGTATTATCCCGTATTGACGCCGGGCAAGAGCAACTCGGTCGCCGCATACACTATTCTCAGAATGACTTGGTTGAGTACTCACCAGTCACAGAAAAGCATCTTACGGATGGCATGACAGTAAGAGAATTATGCAGTGCTGCCATAACCATGAGTGATAACACTGCGGCCAACTTACTTCTGACAACGATCGGAGGACCGAAGGAGCTAACCGCTTTTTTGCACAACATGGGGGATCATGTAACTCGCCTTGATCGTTGGGAACCGGAGCTGAATGAAGCCATACCAAACGACGAGCGTGACACCACGATGCCTGTAGCAATGGCAACAACGTTGCGCAAACTATTAACTGGCGAACTACTTACTCTAGCTTCCCGGCAACAATTAATAGACTGGATGGAGGCGGATAAAGTTGCAGGACCACTTCTGCGCTCGGCCCTTCCGGCTGGCTGGTTTATTGCTGATAAATCTGGAGCCGGTGAGCGTGGGTCTCGCGGTATCATTGCAGCACTGGGGCCAGATGGTAAGCCCTCCCGTATCGTAGTTATCTACACGACGGGGAGTCAGGCAACTATGGATGAACGAAATAGACAGATCGCTGAGATAGGTGCCTCACTGATTAAGCATTGGTAACTGTCAGACCAAGTTTACTCATATATACTTTAGATTGATTTAAAACTTCATTTTTAATTTAAAAGGATCTAGGTGAAGATCCTTTTTGATAATCTCATGACCAAAATCCCTTAACGTGAGTTTTCGTTCCACTGAGCGTCAGACCCCGTAGAAAAGATCAAAGGATCTTCTTGAGATCCTTTTTTTCTGCGCGTAATCTGCTGCTTGCAAACAAAAAAACCACCGCTACCAGCGGTGGTTTGTTTGCCGGATCAAGAGCTACCAACTCTTTTTCCGAAGGTAACTGGCTTCAGCAGAGCGCAGATACCAAATACTGTCCTTCTAGTGTAGCCGTAGTTAGGCCACCACTTCAAGAACTCTGTAGCACCGCCTACATACCTCGCTCTGCTAATCCTGTTACCAGTGGCTGCTGCCAGTGGCGATAAGTCGTGTCTTACCGGGTTGGACTCAAGACGATAGTTACCGGATAAGGCGCAGCGGTCGGGCTGAACGGGGGGTTCGTGCACACAGCCCAGCTTGGAGCGAACGACCTACACCGAACTGAGATACCTACAGCGTGAGCTATGAGAAAGCGCCACGCTTCCCGAAGGGAGAAAGGCGGACAGGTATCCGGTAAGCGGCAGGGTCGGAACAGGAGAGCGCACGAGGGAGCTTCCAGGGGGAAACGCCTGGTATCTTTATAGTCCTGTCGGGTTTCGCCACCTCTGACTTGAGCGTCGATTTTTGTGATGCTCGTCAGGGGGGCGGAGCCTATGGAAAAACGCCAGCAACGCGGCCTTTTTACGGTTCCTGGCCTTTTGCTGGCCTTTTGCTCACATGTTCTTTCCTGCGTTATCCCCTGATTCTGTGGATAACCGTATTACCGCCTTTGAGTGAGCTGATACCGCTCGCCGCAGCCGAACGACCGAGCGCAGCGAGTCAGTGAGCGAGGAAGCGGAAGAGCGCCCAATACGCAAACCGCCTCTCCCCGCGCGTTGGCCGATTCATTAATGCAGCTGGCACGACAGGTTTCCCGACTGGAAAGCGGGCAGTGAGCGCAACGCAATTAATGTGAGTTACCTCACTCATTAGGCACCCCAGGCTTTACACTTTATGCTTCCGGCTCCTATGTTGTGTGGAATTGTGAGCGGATAACAATTTCACACAGGAAACAGCTATGACCATGATTACGCCAAGCGCGCAATTAACCCTCACTAAAGGGAACAAAAGCTGGAGAAGATGATATTGAAGGAGCATTTTTGGGCTTGGCTGGAGCTAGTGGAGGTCAACACATCAATGCTATTTTGGTTTAGTCGTCCAGGCGGATCACAAAATTTGTGTCGTTTGACAAGATGGTTCATTTAGGCAACTGGTCAGATCAGCCCACTTGTAAGCAGTAGCGGCGGCGCTCGAAGTGTGACTCTTATTAGCAGACAGGAACGAGGACATTATTATCATCTGCTGCTTGGTGCACGATAACTTGTGCGTTTGTCAAGCAAGGTAAGTGAACGACCCGGTCATACCTTCTTAAGTTCGCCCTTCCTCCCTTTATTTCAGATTCAATCTGACTTACCTATTCTACCGAAGCACTAGTGGATCCCCCGGGAAAAAAATGGACAAGAAGTACTCCATTGGGCTCGATATCGGCACAAACAGCGTCGGCTGGGCCGTCATTACGGACGAGTACAAGGTGCCGAGCAAAAAATTCAAAGTTCTGGGCAATACCGATCGCCACAGCATAAAGAAGAACCTCATTGGCGCCCTCCTGTTCGACTCCGGGGAGACGGCCGAAGCCACGCGGCTCAAAAGAACAGCACGGCGCAGATATACCCGCAGAAAGAATCGGATCTGCTACCTGCAGGAGATCTTTAGTAATGAGATGGCTAAGGTGGATGACTCTTTCTTCCATAGGCTGGAGGAGTCCTTTTTGGTGGAGGAGGATAAAAAGCACGAGCGCCACCCAATCTTTGGCAATATCGTGGACGAGGTGGCGTACCATGAAAAGTACCCAACCATATATCATCTGAGGAAGAAGCTTGTAGACAGTACTGATAAGGCTGACTTGCGGTTGATCTATCTCGCGCTGGCGCATATGATCAAATTTCGGGGACACTTCCTCATCGAGGGGGACCTGAACCCAGACAACAGCGATGTCGACAAACTCTTTATCCAACTGGTTCAGACTTACAATCAGCTTTTCGAAGAGAACCCGATCAACGCATCCGGAGTTGACGCCAAAGCAATCCTGAGCGCTAGGCTGTCCAAATCCCGGCGGCTCGAAAACCTCATCGCACAGCTCCCTGGGGAGAAGAAGAACGGCCTGTTTGGTAATCTTATCGCCCTGTCACTCGGGCTGACCCCCAACTTTAAATCTAACTTCGACCTGGCCGAAGATGCCAAGCTTCAACTGAGCAAAGACACCTACGATGATGATCTCGACAATCTGCTGGCCCAGATCGGCGACCAGTACGCAGACCTTTTTTTGGCGGCAAAGAACCTGTCAGACGCCATTCTGCTGAGTGATATTCTGCGAGTGAACACGGAGATCACCAAAGCTCCGCTGAGCGCTAGTATGATCAAGCGCTATGATGAGCACCACCAAGACTTGACTTTGCTGAAGGCCCTTGTCAGACAGCAACTGCCTGAGAAGTACAAGGAAATTTTCTTCGATCAGTCTAAAAATGGCTACGCCGGATACATTGACGGCGGAGCAAGCCAGGAGGAATTTTACAAATTTATTAAGCCCATCTTGGAAAAAATGGACGGCACCGAGGAGCTGCTGGTAAAGCTTAACAGAGAAGATCTGTTGCGCAAACAGCGCACTTTCGACAATGGAAGCATCCCCCACCAGATTCACCTGGGCGAACTGCACGCTATCCTCAGGCGGCAAGAGGATTTCTACCCCTTTTTGAAAGATAACAGGGAAAAGATTGAGAAAATCCTCACATTTCGGATACCCTACTATGTAGGCCCCCTCGCCCGGGGAAATTCCAGATTCGCGTGGATGACTCGCAAATCAGAAGAGACCATCACTCCCTGGAACTTCGAGGAAGTCGTGGATAAGGGGGCCTCTGCCCAGTCCTTCATCGAAAGGATGACTAACTTTGATAAAAATCTGCCTAACGAAAAGGTGCTTCCTAAACACTCTCTGCTGTACGAGTACTTCACAGTTTATAACGAGCTCACCAAGGTCAAATACGTCACAGAAGGGATGAGAAAGCCAGCATTCCTGTCTGGAGAGCAGAAGAAAGCTATCGTGGACCTCCTCTTCAAGACGAACCGGAAAGTTACCGTGAAACAGCTCAAAGAAGACTATTTCAAAAAGATTGAATGTTTCGACTCTGTTGAAATCAGCGGAGTGGAGGATCGCTTCAACGCATCCCTGGGAACGTATCACGATCTCCTGAAAATCATTAAAGACAAGGACTTCCTGGACAATGAGGAGAACGAGGACATTCTTGAGGACATTGTCCTCACCCTTACGTTGTTTGAAGATAGGGAGATGATTGAAGAACGCTTGAAAACTTACGCTCATCTCTTCGACGACAAAGTCATGAAACAGCTCAAGAGGCGCCGATATACAGGATGGGGGCGGCTGTCAAGAAAACTGATCAATGGGATCCGAGACAAGCAGAGTGGAAAGACAATCCTGGATTTTCTTAAGTCCGATGGATTTGCCAACCGGAACTTCATGCAGTTGATCCATGATGACTCTCTCACCTTTAAGGAGGACATCCAGAAAGCACAAGTTTCTGGCCAGGGGGACAGTCTTCACGAGCACATCGCTAATCTTGCAGGTAGCCCAGCTATCAAAAAGGGAATACTGCAGACCGTTAAGGTCGTGGATGAACTCGTCAAAGTAATGGGAAGGCATAAGCCCGAGAATATCGTTATCGAGATGGCCCGAGAGAACCAAACTACCCAGAAGGGACAGAAGAACAGTAGGGAAAGGATGAAGAGGATTGAAGAGGGTATAAAAGAACTGGGGTCCCAAATCCTTAAGGAACACCCAGTTGAAAACACCCAGCTTCAGAATGAGAAGCTCTACCTGTACTACCTGCAGAACGGCAGGGACATGTACGTGGATCAGGAACTGGACATCAATCGGCTCTCCGACTACGACGTGGATCATATCGTGCCCCAGTCTTTTCTCAAAGATGATTCTATTGATAATAAAGTGTTGACAAGATCCGATAAAAATAGAGGGAAGAGTGATAACGTCCCCTCAGAAGAAGTTGTCAAGAAAATGAAAAATTATTGGCGGCAGCTGCTGAACGCCAAACTGATCACACAACGGAAGTTCGATAATCTGACTAAGGCTGAACGAGGTGGCCTGTCTGAGTTGGATAAAGCCGGCTTCATCAAAAGGCAGCTTGTTGAGACACGCCAGATCACCAAGCACGTGGCCCAAATTCTCGATTCACGCATGAACACCAAGTACGATGAAAATGACAAACTGATTCGAGAGGTGAAAGTTATTACTCTGAAGTCTAAGCTGGTCTCAGATTTCAGAAAGGACTTTCAGTTTTATAAGGTGAGAGAGATCAACAATTACCACCATGCGCATGATGCCTACCTGAATGCAGTGGTAGGCACTGCACTTATCAAAAAATATCCCAAGCTTGAATCTGAATTTGTTTACGGAGACTATAAAGTGTACGATGTTAGGAAAATGATCGCAAAGTCTGAGCAGGAAATAGGCAAGGCCACCGCTAAGTACTTCTTTTACAGCAATATTATGAATTTTTTCAAGACCGAGATTACACTGGCCAATGGAGAGATTCGGAAGCGACCACTTATCGAAACAAACGGAGAAACAGGAGAAATCGTGTGGGACAAGGGTAGGGATTTCGCGACAGTCCGGAAGGTCCTGTCCATGCCGCAGGTGAACATCGTTAAAAAGACCGAAGTACAGACCGGAGGCTTCTCCAAGGAAAGTATCCTCCCGAAAAGGAACAGCGACAAGCTGATCGCACGCAAAAAAGATTGGGACCCCAAGAAATACGGCGGATTCGATTCTCCTACAGTCGCTTACAGTGTACTGGTTGTGGCCAAAGTGGAGAAAGGGAAGTCTAAAAAACTCAAAAGCGTCAAGGAACTGCTGGGCATCACAATCATGGAGCGATCAAGCTTCGAAAAAAACCCCATCGACTTTCTCGAGGCGAAAGGATATAAAGAGGTCAAAAAAGACCTCATCATTAAGCTTCCCAAGTACTCTCTCTTTGAGCTTGAAAACGGCCGGAAACGAATGCTCGCTAGTGCGGGCGAGCTGCAGAAAGGTAACGAGCTGGCACTGCCCTCTAAATACGTTAATTTCTTGTATCTGGCCAGCCACTATGAAAAGCTCAAAGGGTCTCCCGAAGATAATGAGCAGAAGCAGCTGTTCGTGGAACAACACAAACACTACCTTGATGAGATCATCGAGCAAATAAGCGAATTCTCCAAAAGAGTGATCCTCGCCGACGCTAACCTCGATAAGGTGCTTTCTGCTTACAATAAGCACAGGGATAAGCCCATCAGGGAGCAGGCAGAAAACATTATCCACTTGTTTACTCTGACCAACTTGGGCGCGCCTGCAGCCTTCAAGTACTTCGACACCACCATAGACAGAAAGCGGTACACCTCTACAAAGGAGGTCCTGGACGCCACACTGATTCATCAGTCAATTACGGGGCTCTATGAAACAAGAATCGACCTCTCTCAGCTCGGTGGAGACAGCAGGGCTGACCCCAAGAAGAAGAGGAAGGTGTGATTTTGGACCTCGAGTCATTGGACCTCGAGTCATGTAATTAGTTATGTCACGCTTACATTCACGCCCTCCCCCCACATCCGCTCTAACCGAAAAGGAAGGAGTTAGACAACCTGAAGTCTAGGTCCCTATTTATTTTTTTATAGTTATGTTAGTATTAAGAACGTTATTTATATTTCAAATTTTTCTTTTTTTTCTGTACAGACGCGTACTTAACGTTACTGAAATCATCAAACAGCTTGACGAATCTGGATATAAGATCGTTGGTGTCGATGTCAGCTCCGGAGTTGAGACAAATGGTGTTCAGGATCTCGATAAGATACGTTCATTTGTCCAAGCAGCAAAGAGTGCCTTCTAGTGATTTAATAGCTCCATGTCAACAAGAATAAAACGCGTTTTCGGGTTTACCTCTTCCAGATACAGCTCATCTGCAATGCATTAATGCATTGACTGCAACCTAGTAACGCCTTCAGGCTCCGGCGAAGAGAAGAATAGCTTAGCAGAGCTATTTTCATTTTCGGGAGACGAGATCAAGCAGATCAACGGTCGTCAAGAGACCTACGAGACTGAGGAATCCGCTCTTGGCTCCACGCGACTATATATTTGTCTCTAATTGTACTTTGACATGCTCCTCTTCTTTACTCTGATAGCTTGACTATGAAAATTCCGTCACCAGCCCTGGGTTCGCAAAGATAATTGCATGTTTCTTCCTTGAACTCTCAAGCCTACAGGACACACATTCATCGTAGGTATAAACCTCGAAATCATTCCTACTAAGATGGTATACAATAGTAACCATGGTTGCCTAGTGAATGCTCCGTAACACCCAATACGCCGGCCGAAACTTTTTTACAACTCTCCTATGAGTCGTTTACCCAGAATGCACAGGTACACTTGTTTAGAGGTAATCCTTCTTTCTAGAGTACGCATGTAACATTATACTGAAAACCTTGCTTGAGAAGGTTTTGGGACGCTCGAAGGCTTTAATTTGCGGCCGGTACCCAATTCGCCCTATAGTGAGTCGTATTACGCGCGCTCACTGGCCGTCGTTTTACAACGTCGTGACTGGGAAAACCCTGGCGTTACCCAACTTAATCGCCTTGCAGCACATCCCCCTTTCGCCAGCTGGCGTAATAGCGAAGAGGCCCGCACCGATCGCCCTTCCCAACAGTTGCGCAGCCTGAATGGCGAATGGCGCGACGCGCCCTGTAGCGGCGCATTAAGCGCGGCGGGTGTGGTGGTTACGCGCAGCGTGACCGCTACACTTGCCAGCGCCCTAGCGCCCGCTCCTTTCGCTTTCTTCCCTTCCTTTCTCGCCACGTTCGCCGGCTTTCCCCGTCAAGCTCTAAATCGGGGGCTCCCTTTAGGGTTCCGATTTAGTGCTTTACGGCACCTCGACCCCAAAAAACTTGATTAGGGTGATGGTTCACGTAGTGGGCCATCGCCCTGATAGACGGTTTTTCGCCCTTTGACGTTGGAGTCCACGTTCTTTAATAGTGGACTCTTGTTCCAAACTGGAACAACACTCAACCCTATCTCGGTCTATTCTTTTGATTTATAAGGGATTTTGCCGATTTCGGCCTATTGGTTAAAAAATGAGCTGATTTAACAAAAATTTAACGCGAATTTTAACAAAATATTAACGTTTACAATTTCCTGATGCGGTATTTTCTCCTTACGCATCTGTGCGGTATTTCACACCGCATATCGACGGTCGAGGAGAACTTCTAGTATATCCACATACCTAATATTATTGCCTTATTAAAAATGGAATCCCAACAATTACATCAAAATCCACATTCTCTTCAAAATCAATTGTCCTGTACTTCCTTGTTCATGTGTGTTCAAAAACGTTATATTTATAGGATAATTATACTCTATTTCTCAACAAGTAATTGGTTGTTTGGCCGAGCGGTCTAAGGCGCCTGATTCAAGAAATATCTTGACCGCAGTTAACTGTGGGAATACTCAGGTATCGTAAGATGCAAGAGTTCGAATCTCTTAGCAACCATTATTTTTTTCCTCAACATAACGAGAACACACAGGGGCGCTATCGCACAGAATCAAATTCGATGACTGGAAATTTTTTGTTAATTTCAGAGGTCGCCTGACGCATATACCTTTTTCAACTGAAAAATTGGGAGAAAAAGGAAAGGTGAGAGGCCGGAACCGGCTTTTCATATAGAATAGAGAAGCGTTCATGACTAAATGCTTGCATCACAATACTTGAAGTTGACAATATTATTTAAGGACCTATTGTTTTTTCCAATAGGTGGTTAGCAATCGTCTTACTTTCTAACTTTTCTTACCTTTTACATTTCAGCAATATATATATATATTTCAAGGATATACCATTCTAATGTCTGCCCCTATGTCTGCCCCTAAGAAGATCGTCGTTTTGCCAGGTGACCACGTTGGTCAAGAAATCACAGCCGAAGCCATTAAGGTTCTTAAAGCTATTTCTGATGTTCGTTCCAATGTCAAGTTCGATTTCGAAAATCATTTAATTGGTGGTGCTGCTATCGATGCTACAGGTGTCCCACTTCCAGATGAGGCGCTGGAAGCCTCCAAGAAGGTTGATGCCGTTTTGTTAGGTGCTGTGGGTGGTCCTAAATGGGGTACCGGTAGTGTTAGACCTGAACAAGGTTTACTAAAAATCCGTAAAGAACTTCAATTGTACGCCAACTTAAGACCATGTAACTTTGCATCCGACTCTCTTTTAGACTTATCTCCAATCAAGCCACAATTTGCTAAAGGTACTGACTTCGTTGTTGTCAGAGAATTAGTGGGAGGTATTTACTTTGGTAAGAGAAAGGAAGACGATGGTGATGGTGTCGCTTGGGATAGTGAACAATACACCGTTCCAGAAGTGCAAAGAATCACAAGAATGGCCGCTTTCATGGCCCTACAACATGAGCCACCATTGCCTATTTGGTCCTTGGATAAAGCTAATGTTTTGGCCTCTTCAAGATTATGGAGAAAAACTGTGGAGGAAACCATCAAGAACGAATTCCCTACATTGAAGGTTCAACATCAATTGATTGATTCTGCCGCCATGATCCTAGTTAAGAACCCAACCCACCTAAATGGTATTATAATCACCAGCAACATGTTTGGTGATATCATCTCCGATGAAGCCTCCGTTATCCCAGGTTCCTTGGGTTTGTTGCCATCTGCGTCCTTGGCCTCTTTGCCAGACAAGAACACCGCATTTGGTTTGTACGAACCATGCCACGGTTCTGCTCCAGATTTGCCAAAGAATAAGGTTGACCCTATCGCCACTATCTTGTCTGCTGCAATGATGTTGAAATTGTCATTGAACTTGCCTGAAGAAGGTAAGGCCATTGAAGATGCAGTTAAAAAGGTTTTGGATGCAGGTATCAGAACTGGTGATTTAGGTGGTTCCAACAGTACCACCGAAGTCGGTGATGCTGTCGCCGAAGAAGTTAAGAAAATCCTTGCTTAAAAAGATTCTCTTTTTTTATGATATTTGTACATAAACTTTATAAATGAAATTCATAATAGAAACGACACGAAATTACAAAATGGAATATGTTCATAGGGTAGACGAAACTATATACGCAATCTACATACATTTATCAAGAAGGAGAAAAAGGAGGATAGTAAAGGAATACAGGTAAGCAAATTGATACTAATGGCTCAACGTGATAAGGAAAAAGAATTGCACTTTAACATTAATATTGACAAGGAGGAGGGCACCACACAAAAAGTTAGGTGTAACAGAAAATCATGAAACTACGATTCCTAATTTGATATTGGAGGATTTTCTCTAAAAAAAAAAAAATACAACAAATAAAAAACACTCAATGACCTGACCATTTGATGGAGTTTAAGTCAATACCTTCTTGAACCATTTCCCATAATGGTGAAAGTTCCCTCAAGAATTTTACTCTGTCAGAAACGGCCTTACGACGTAGTCGATATGGTGCACTCTCAGTACAATCTGCTCTGATGCCGCATAGTTAAGCCAGCCCCGACACCCGCCAACACCCGCTGACGCGCCCTGACGGGCTTGTCTGCTCCCGGCATCCGCTTACAGACAAGCTGTGACCGTCTCCGGGAGCTGCATGTGTCAGAGGTTTTCACCGTCATCACCGAAACGCGCGA

>p426-SNR52p-gRNA.clr-2.Y-SUP4t

GACGAAAGGGCCTCGTGATACGCCTATTTTTATAGGTTAATGTCATGATAATAATGGTTTCTTAGTATGATCCAATATCAAAGGAAATGATAGCATTGAAGGATGAGACTAATCCAATTGAGGAGTGGCAGCATATAGAACAGCTAAAGGGTAGTGCTGAAGGAAGCATACGATACCCCGCATGGAATGGGATAATATCACAGGAGGTACTAGACTACCTTTCATCCTACATAAATAGACGCATATAAGTACGCATTTAAGCATAAACACGCACTATGCCGTTCTTCTCATGTATATATATATACAGGCAACACGCAGATATAGGTGCGACGTGAACAGTGAGCTGTATGTGCGCAGCTCGCGTTGCATTTTCGGAAGCGCTCGTTTTCGGAAACGCTTTGAAGTTCCTATTCCGAAGTTCCTATTCTCTAGAAAGTATAGGAACTTCAGAGCGCTTTTGAAAACCAAAAGCGCTCTGAAGACGCACTTTCAAAAAACCAAAAACGCACCGGACTGTAACGAGCTACTAAAATATTGCGAATACCGCTTCCACAAACATTGCTCAAAAGTATCTCTTTGCTATATATCTCTGTGCTATATCCCTATATAACCTACCCATCCACCTTTCGCTCCTTGAACTTGCATCTAAACTCGACCTCTACATTTTTTATGTTTATCTCTAGTATTACTCTTTAGACAAAAAAATTGTAGTAAGAACTATTCATAGAGTGAATCGAAAACAATACGAAAATGTAAACATTTCCTATACGTAGTATATAGAGACAAAATAGAAGAAACCGTTCATAATTTTCTGACCAATGAAGAATCATCAACGCTATCACTTTCTGTTCACAAAGTATGCGCAATCCACATCGGTATAGAATATAATCGGGGATGCCTTTATCTTGAAAAAATGCACCCGCAGCTTCGCTAGTAATCAGTAAACGCGGGAAGTGGAGTCAGGCTTTTTTTATGGAAGAGAAAATAGACACCAAAGTAGCCTTCTTCTAACCTTAACGGACCTACAGTGCAAAAAGTTATCAAGAGACTGCATTATAGAGCGCACAAAGGAGAAAAAAAGTAATCTAAGATGCTTTGTTAGAAAAATAGCGCTCTCGGGATGCATTTTTGTAGAACAAAAAAGAAGTATAGATTCTTTGTTGGTAAAATAGCGCTCTCGCGTTGCATTTCTGTTCTGTAAAAATGCAGCTCAGATTCTTTGTTTGAAAAATTAGCGCTCTCGCGTTGCATTTTTGTTTTACAAAAATGAAGCACAGATTCTTCGTTGGTAAAATAGCGCTTTCGCGTTGCATTTCTGTTCTGTAAAAATGCAGCTCAGATTCTTTGTTTGAAAAATTAGCGCTCTCGCGTTGCATTTTTGTTCTACAAAATGAAGCACAGATGCTTCGTTCAGGTGGCACTTTTCGGGGAAATGTGCGCGGAACCCCTATTTGTTTATTTTTCTAAATACATTCAAATATGTATCCGCTCATGAGACAATAACCCTGATAAATGCTTCAATAATATTGAAAAAGGAAGAGTATGAGTATTCAACATTTCCGTGTCGCCCTTATTCCCTTTTTTGCGGCATTTTGCCTTCCTGTTTTTGCTCACCCAGAAACGCTGGTGAAAGTAAAAGATGCTGAAGATCAGTTGGGTGCACGAGTGGGTTACATCGAACTGGATCTCAACAGCGGTAAGATCCTTGAGAGTTTTCGCCCCGAAGAACGTTTTCCAATGATGAGCACTTTTAAAGTTCTGCTATGTGGCGCGGTATTATCCCGTATTGACGCCGGGCAAGAGCAACTCGGTCGCCGCATACACTATTCTCAGAATGACTTGGTTGAGTACTCACCAGTCACAGAAAAGCATCTTACGGATGGCATGACAGTAAGAGAATTATGCAGTGCTGCCATAACCATGAGTGATAACACTGCGGCCAACTTACTTCTGACAACGATCGGAGGACCGAAGGAGCTAACCGCTTTTTTGCACAACATGGGGGATCATGTAACTCGCCTTGATCGTTGGGAACCGGAGCTGAATGAAGCCATACCAAACGACGAGCGTGACACCACGATGCCTGTAGCAATGGCAACAACGTTGCGCAAACTATTAACTGGCGAACTACTTACTCTAGCTTCCCGGCAACAATTAATAGACTGGATGGAGGCGGATAAAGTTGCAGGACCACTTCTGCGCTCGGCCCTTCCGGCTGGCTGGTTTATTGCTGATAAATCTGGAGCCGGTGAGCGTGGGTCTCGCGGTATCATTGCAGCACTGGGGCCAGATGGTAAGCCCTCCCGTATCGTAGTTATCTACACGACGGGGAGTCAGGCAACTATGGATGAACGAAATAGACAGATCGCTGAGATAGGTGCCTCACTGATTAAGCATTGGTAACTGTCAGACCAAGTTTACTCATATATACTTTAGATTGATTTAAAACTTCATTTTTAATTTAAAAGGATCTAGGTGAAGATCCTTTTTGATAATCTCATGACCAAAATCCCTTAACGTGAGTTTTCGTTCCACTGAGCGTCAGACCCCGTAGAAAAGATCAAAGGATCTTCTTGAGATCCTTTTTTTCTGCGCGTAATCTGCTGCTTGCAAACAAAAAAACCACCGCTACCAGCGGTGGTTTGTTTGCCGGATCAAGAGCTACCAACTCTTTTTCCGAAGGTAACTGGCTTCAGCAGAGCGCAGATACCAAATACTGTCCTTCTAGTGTAGCCGTAGTTAGGCCACCACTTCAAGAACTCTGTAGCACCGCCTACATACCTCGCTCTGCTAATCCTGTTACCAGTGGCTGCTGCCAGTGGCGATAAGTCGTGTCTTACCGGGTTGGACTCAAGACGATAGTTACCGGATAAGGCGCAGCGGTCGGGCTGAACGGGGGGTTCGTGCACACAGCCCAGCTTGGAGCGAACGACCTACACCGAACTGAGATACCTACAGCGTGAGCTATGAGAAAGCGCCACGCTTCCCGAAGGGAGAAAGGCGGACAGGTATCCGGTAAGCGGCAGGGTCGGAACAGGAGAGCGCACGAGGGAGCTTCCAGGGGGAAACGCCTGGTATCTTTATAGTCCTGTCGGGTTTCGCCACCTCTGACTTGAGCGTCGATTTTTGTGATGCTCGTCAGGGGGGCGGAGCCTATGGAAAAACGCCAGCAACGCGGCCTTTTTACGGTTCCTGGCCTTTTGCTGGCCTTTTGCTCACATGTTCTTTCCTGCGTTATCCCCTGATTCTGTGGATAACCGTATTACCGCCTTTGAGTGAGCTGATACCGCTCGCCGCAGCCGAACGACCGAGCGCAGCGAGTCAGTGAGCGAGGAAGCGGAAGAGCGCCCAATACGCAAACCGCCTCTCCCCGCGCGTTGGCCGATTCATTAATGCAGCTGGCACGACAGGTTTCCCGACTGGAAAGCGGGCAGTGAGCGCAACGCAATTAATGTGAGTTACCTCACTCATTAGGCACCCCAGGCTTTACACTTTATGCTTCCGGCTCCTATGTTGTGTGGAATTGTGAGCGGATAACAATTTCACACAGGAAACAGCTATGACCATGATTACGCCAAGCGCGCAATTAACCCTCACTAAAGGGAACAAAAGCTGGAGCTtctttgaaaagataatgtatgattatgctttcactcatatttatacagaaacttgatgttttctttcgagtatatacaaggtgattacatgtacgtttgaagtacaactctagattttgtagtgccctcttgggctagcggtaaaggtgcgcattttttcacaccctacaatgttctgttcaaaagattttggtcaaacgctgtagaagtgaaagttggtgcgcatgtttcggcgttcgaaacttctccgcagtgaaagataaatgatcGAGTGCCCTAGTCGGTGTGAGTTTTAGAGCTAGAAATAGCAAGTTAAAATAAGGCTAGTCCGTTATCAACTTGAAAAAGTGGCACCGAGTCGGTGGTGCTTTTTTTGTTTTTTATGTCTTCGAGTCATGTAATTAGTTATGTCACGCTTACATTCACGCCCTCCCCCCACATCCGCTCTAACCGAAAAGGAAGGAGTTAGACAACCTGAAGTCTAGGTCCCTATTTATTTTTTTATAGTTATGTTAGTATTAAGAACGTTATTTATATTTCAAATTTTTCTTTTTTTTCTGTACAGACGCGTGTACGCATGTAACATTATACTGAAAACCTTGCTTGAGAAGGTTTTGGGACGCTCGAAGGCTTTAATTTGCGGCCGGTACCCAATTCGCCCTATAGTGAGTCGTATTACGCGCGCTCACTGGCCGTCGTTTTACAACGTCGTGACTGGGAAAACCCTGGCGTTACCCAACTTAATCGCCTTGCAGCACATCCCCCTTTCGCCAGCTGGCGTAATAGCGAAGAGGCCCGCACCGATCGCCCTTCCCAACAGTTGCGCAGCCTGAATGGCGAATGGCGCGACGCGCCCTGTAGCGGCGCATTAAGCGCGGCGGGTGTGGTGGTTACGCGCAGCGTGACCGCTACACTTGCCAGCGCCCTAGCGCCCGCTCCTTTCGCTTTCTTCCCTTCCTTTCTCGCCACGTTCGCCGGCTTTCCCCGTCAAGCTCTAAATCGGGGGCTCCCTTTAGGGTTCCGATTTAGTGCTTTACGGCACCTCGACCCCAAAAAACTTGATTAGGGTGATGGTTCACGTAGTGGGCCATCGCCCTGATAGACGGTTTTTCGCCCTTTGACGTTGGAGTCCACGTTCTTTAATAGTGGACTCTTGTTCCAAACTGGAACAACACTCAACCCTATCTCGGTCTATTCTTTTGATTTATAAGGGATTTTGCCGATTTCGGCCTATTGGTTAAAAAATGAGCTGATTTAACAAAAATTTAACGCGAATTTTAACAAAATATTAACGTTTACAATTTCCTGATGCGGTATTTTCTCCTTACGCATCTGTGCGGTATTTCACACCGCATAGGGTAATAACTGATATAATTAAATTGAAGCTCTAATTTGTGAGTTTAGTATACATGCATTTACTTATAATACAGTTTTTTAGTTTTGCTGGCCGCATCTTCTCAAATATGCTTCCCAGCCTGCTTTTCTGTAACGTTCACCCTCTACCTTAGCATCCCTTCCCTTTGCAAATAGTCCTCTTCCAACAATAATAATGTCAGATCCTGTAGAGACCACATCATCCACGGTTCTATACTGTTGACCCAATGCGTCTCCCTTGTCATCTAAACCCACACCGGGTGTCATAATCAACCAATCGTAACCTTCATCTCTTCCACCCATGTCTCTTTGAGCAATAAAGCCGATAACAAAATCTTTGTCGCTCTTCGCAATGTCAACAGTACCCTTAGTATATTCTCCAGTAGATAGGGAGCCCTTGCATGACAATTCTGCTAACATCAAAAGGCCTCTAGGTTCCTTTGTTACTTCTTCTGCCGCCTGCTTCAAACCGCTAACAATACCTGGGCCCACCACACCGTGTGCATTCGTAATGTCTGCCCATTCTGCTATTCTGTATACACCCGCAGAGTACTGCAATTTGACTGTATTACCAATGTCAGCAAATTTTCTGTCTTCGAAGAGTAAAAAATTGTACTTGGCGGATAATGCCTTTAGCGGCTTAACTGTGCCCTCCATGGAAAAATCAGTCAAGATATCCACATGTGTTTTTAGTAAACAAATTTTGGGACCTAATGCTTCAACTAACTCCAGTAATTCCTTGGTGGTACGAACATCCAATGAAGCACACAAGTTTGTTTGCTTTTCGTGCATGATATTAAATAGCTTGGCAGCAACAGGACTAGGATGAGTAGCAGCACGTTCCTTATATGTAGCTTTCGACATGATTTATCTTCGTTTCCTGCAGGTTTTTGTTCTGTGCAGTTGGGTTAAGAATACTGGGCAATTTCATGTTTCTTCAACACTACATATGCGTATATATACCAATCTAAGTCTGTGCTCCTTCCTTCGTTCTTCCTTCTGTTCGGAGATTACCGAATCAAAAAAATTTCAAAGAAACCGAAATCAAAAAAAAGAATAAAAAAAAAATGATGAATTGAATTGAAAAGCTGTGGTATGGTGCACTCTCAGTACAATCTGCTCTGATGCCGCATAGTTAAGCCAGCCCCGACACCCGCCAACACCCGCTGACGCGCCCTGACGGGCTTGTCTGCTCCCGGCATCCGCTTACAGACAAGCTGTGACCGTCTCCGGGAGCTGCATGTGTCAGAGGTTTTCACCGTCATCACCGAAACGCGCGA

>p426-SNR52p-gRNA.csr-1.Y-SUP4t

GACGAAAGGGCCTCGTGATACGCCTATTTTTATAGGTTAATGTCATGATAATAATGGTTTCTTAGTATGATCCAATATCAAAGGAAATGATAGCATTGAAGGATGAGACTAATCCAATTGAGGAGTGGCAGCATATAGAACAGCTAAAGGGTAGTGCTGAAGGAAGCATACGATACCCCGCATGGAATGGGATAATATCACAGGAGGTACTAGACTACCTTTCATCCTACATAAATAGACGCATATAAGTACGCATTTAAGCATAAACACGCACTATGCCGTTCTTCTCATGTATATATATATACAGGCAACACGCAGATATAGGTGCGACGTGAACAGTGAGCTGTATGTGCGCAGCTCGCGTTGCATTTTCGGAAGCGCTCGTTTTCGGAAACGCTTTGAAGTTCCTATTCCGAAGTTCCTATTCTCTAGAAAGTATAGGAACTTCAGAGCGCTTTTGAAAACCAAAAGCGCTCTGAAGACGCACTTTCAAAAAACCAAAAACGCACCGGACTGTAACGAGCTACTAAAATATTGCGAATACCGCTTCCACAAACATTGCTCAAAAGTATCTCTTTGCTATATATCTCTGTGCTATATCCCTATATAACCTACCCATCCACCTTTCGCTCCTTGAACTTGCATCTAAACTCGACCTCTACATTTTTTATGTTTATCTCTAGTATTACTCTTTAGACAAAAAAATTGTAGTAAGAACTATTCATAGAGTGAATCGAAAACAATACGAAAATGTAAACATTTCCTATACGTAGTATATAGAGACAAAATAGAAGAAACCGTTCATAATTTTCTGACCAATGAAGAATCATCAACGCTATCACTTTCTGTTCACAAAGTATGCGCAATCCACATCGGTATAGAATATAATCGGGGATGCCTTTATCTTGAAAAAATGCACCCGCAGCTTCGCTAGTAATCAGTAAACGCGGGAAGTGGAGTCAGGCTTTTTTTATGGAAGAGAAAATAGACACCAAAGTAGCCTTCTTCTAACCTTAACGGACCTACAGTGCAAAAAGTTATCAAGAGACTGCATTATAGAGCGCACAAAGGAGAAAAAAAGTAATCTAAGATGCTTTGTTAGAAAAATAGCGCTCTCGGGATGCATTTTTGTAGAACAAAAAAGAAGTATAGATTCTTTGTTGGTAAAATAGCGCTCTCGCGTTGCATTTCTGTTCTGTAAAAATGCAGCTCAGATTCTTTGTTTGAAAAATTAGCGCTCTCGCGTTGCATTTTTGTTTTACAAAAATGAAGCACAGATTCTTCGTTGGTAAAATAGCGCTTTCGCGTTGCATTTCTGTTCTGTAAAAATGCAGCTCAGATTCTTTGTTTGAAAAATTAGCGCTCTCGCGTTGCATTTTTGTTCTACAAAATGAAGCACAGATGCTTCGTTCAGGTGGCACTTTTCGGGGAAATGTGCGCGGAACCCCTATTTGTTTATTTTTCTAAATACATTCAAATATGTATCCGCTCATGAGACAATAACCCTGATAAATGCTTCAATAATATTGAAAAAGGAAGAGTATGAGTATTCAACATTTCCGTGTCGCCCTTATTCCCTTTTTTGCGGCATTTTGCCTTCCTGTTTTTGCTCACCCAGAAACGCTGGTGAAAGTAAAAGATGCTGAAGATCAGTTGGGTGCACGAGTGGGTTACATCGAACTGGATCTCAACAGCGGTAAGATCCTTGAGAGTTTTCGCCCCGAAGAACGTTTTCCAATGATGAGCACTTTTAAAGTTCTGCTATGTGGCGCGGTATTATCCCGTATTGACGCCGGGCAAGAGCAACTCGGTCGCCGCATACACTATTCTCAGAATGACTTGGTTGAGTACTCACCAGTCACAGAAAAGCATCTTACGGATGGCATGACAGTAAGAGAATTATGCAGTGCTGCCATAACCATGAGTGATAACACTGCGGCCAACTTACTTCTGACAACGATCGGAGGACCGAAGGAGCTAACCGCTTTTTTGCACAACATGGGGGATCATGTAACTCGCCTTGATCGTTGGGAACCGGAGCTGAATGAAGCCATACCAAACGACGAGCGTGACACCACGATGCCTGTAGCAATGGCAACAACGTTGCGCAAACTATTAACTGGCGAACTACTTACTCTAGCTTCCCGGCAACAATTAATAGACTGGATGGAGGCGGATAAAGTTGCAGGACCACTTCTGCGCTCGGCCCTTCCGGCTGGCTGGTTTATTGCTGATAAATCTGGAGCCGGTGAGCGTGGGTCTCGCGGTATCATTGCAGCACTGGGGCCAGATGGTAAGCCCTCCCGTATCGTAGTTATCTACACGACGGGGAGTCAGGCAACTATGGATGAACGAAATAGACAGATCGCTGAGATAGGTGCCTCACTGATTAAGCATTGGTAACTGTCAGACCAAGTTTACTCATATATACTTTAGATTGATTTAAAACTTCATTTTTAATTTAAAAGGATCTAGGTGAAGATCCTTTTTGATAATCTCATGACCAAAATCCCTTAACGTGAGTTTTCGTTCCACTGAGCGTCAGACCCCGTAGAAAAGATCAAAGGATCTTCTTGAGATCCTTTTTTTCTGCGCGTAATCTGCTGCTTGCAAACAAAAAAACCACCGCTACCAGCGGTGGTTTGTTTGCCGGATCAAGAGCTACCAACTCTTTTTCCGAAGGTAACTGGCTTCAGCAGAGCGCAGATACCAAATACTGTCCTTCTAGTGTAGCCGTAGTTAGGCCACCACTTCAAGAACTCTGTAGCACCGCCTACATACCTCGCTCTGCTAATCCTGTTACCAGTGGCTGCTGCCAGTGGCGATAAGTCGTGTCTTACCGGGTTGGACTCAAGACGATAGTTACCGGATAAGGCGCAGCGGTCGGGCTGAACGGGGGGTTCGTGCACACAGCCCAGCTTGGAGCGAACGACCTACACCGAACTGAGATACCTACAGCGTGAGCTATGAGAAAGCGCCACGCTTCCCGAAGGGAGAAAGGCGGACAGGTATCCGGTAAGCGGCAGGGTCGGAACAGGAGAGCGCACGAGGGAGCTTCCAGGGGGAAACGCCTGGTATCTTTATAGTCCTGTCGGGTTTCGCCACCTCTGACTTGAGCGTCGATTTTTGTGATGCTCGTCAGGGGGGCGGAGCCTATGGAAAAACGCCAGCAACGCGGCCTTTTTACGGTTCCTGGCCTTTTGCTGGCCTTTTGCTCACATGTTCTTTCCTGCGTTATCCCCTGATTCTGTGGATAACCGTATTACCGCCTTTGAGTGAGCTGATACCGCTCGCCGCAGCCGAACGACCGAGCGCAGCGAGTCAGTGAGCGAGGAAGCGGAAGAGCGCCCAATACGCAAACCGCCTCTCCCCGCGCGTTGGCCGATTCATTAATGCAGCTGGCACGACAGGTTTCCCGACTGGAAAGCGGGCAGTGAGCGCAACGCAATTAATGTGAGTTACCTCACTCATTAGGCACCCCAGGCTTTACACTTTATGCTTCCGGCTCCTATGTTGTGTGGAATTGTGAGCGGATAACAATTTCACACAGGAAACAGCTATGACCATGATTACGCCAAGCGCGCAATTAACCCTCACTAAAGGGAACAAAAGCTGGAGCTtctttgaaaagataatgtatgattatgctttcactcatatttatacagaaacttgatgttttctttcgagtatatacaaggtgattacatgtacgtttgaagtacaactctagattttgtagtgccctcttgggctagcggtaaaggtgcgcattttttcacaccctacaatgttctgttcaaaagattttggtcaaacgctgtagaagtgaaagttggtgcgcatgtttcggcgttcgaaacttctccgcagtgaaagataaatgatcGAGTGGGAGGGTCCCGTCCTGTTTTAGAGCTAGAAATAGCAAGTTAAAATAAGGCTAGTCCGTTATCAACTTGAAAAAGTGGCACCGAGTCGGTGGTGCTTTTTTTGTTTTTTATGTCTTCGAGTCATGTAATTAGTTATGTCACGCTTACATTCACGCCCTCCCCCCACATCCGCTCTAACCGAAAAGGAAGGAGTTAGACAACCTGAAGTCTAGGTCCCTATTTATTTTTTTATAGTTATGTTAGTATTAAGAACGTTATTTATATTTCAAATTTTTCTTTTTTTTCTGTACAGACGCGTGTACGCATGTAACATTATACTGAAAACCTTGCTTGAGAAGGTTTTGGGACGCTCGAAGGCTTTAATTTGCGGCCGGTACCCAATTCGCCCTATAGTGAGTCGTATTACGCGCGCTCACTGGCCGTCGTTTTACAACGTCGTGACTGGGAAAACCCTGGCGTTACCCAACTTAATCGCCTTGCAGCACATCCCCCTTTCGCCAGCTGGCGTAATAGCGAAGAGGCCCGCACCGATCGCCCTTCCCAACAGTTGCGCAGCCTGAATGGCGAATGGCGCGACGCGCCCTGTAGCGGCGCATTAAGCGCGGCGGGTGTGGTGGTTACGCGCAGCGTGACCGCTACACTTGCCAGCGCCCTAGCGCCCGCTCCTTTCGCTTTCTTCCCTTCCTTTCTCGCCACGTTCGCCGGCTTTCCCCGTCAAGCTCTAAATCGGGGGCTCCCTTTAGGGTTCCGATTTAGTGCTTTACGGCACCTCGACCCCAAAAAACTTGATTAGGGTGATGGTTCACGTAGTGGGCCATCGCCCTGATAGACGGTTTTTCGCCCTTTGACGTTGGAGTCCACGTTCTTTAATAGTGGACTCTTGTTCCAAACTGGAACAACACTCAACCCTATCTCGGTCTATTCTTTTGATTTATAAGGGATTTTGCCGATTTCGGCCTATTGGTTAAAAAATGAGCTGATTTAACAAAAATTTAACGCGAATTTTAACAAAATATTAACGTTTACAATTTCCTGATGCGGTATTTTCTCCTTACGCATCTGTGCGGTATTTCACACCGCATAGGGTAATAACTGATATAATTAAATTGAAGCTCTAATTTGTGAGTTTAGTATACATGCATTTACTTATAATACAGTTTTTTAGTTTTGCTGGCCGCATCTTCTCAAATATGCTTCCCAGCCTGCTTTTCTGTAACGTTCACCCTCTACCTTAGCATCCCTTCCCTTTGCAAATAGTCCTCTTCCAACAATAATAATGTCAGATCCTGTAGAGACCACATCATCCACGGTTCTATACTGTTGACCCAATGCGTCTCCCTTGTCATCTAAACCCACACCGGGTGTCATAATCAACCAATCGTAACCTTCATCTCTTCCACCCATGTCTCTTTGAGCAATAAAGCCGATAACAAAATCTTTGTCGCTCTTCGCAATGTCAACAGTACCCTTAGTATATTCTCCAGTAGATAGGGAGCCCTTGCATGACAATTCTGCTAACATCAAAAGGCCTCTAGGTTCCTTTGTTACTTCTTCTGCCGCCTGCTTCAAACCGCTAACAATACCTGGGCCCACCACACCGTGTGCATTCGTAATGTCTGCCCATTCTGCTATTCTGTATACACCCGCAGAGTACTGCAATTTGACTGTATTACCAATGTCAGCAAATTTTCTGTCTTCGAAGAGTAAAAAATTGTACTTGGCGGATAATGCCTTTAGCGGCTTAACTGTGCCCTCCATGGAAAAATCAGTCAAGATATCCACATGTGTTTTTAGTAAACAAATTTTGGGACCTAATGCTTCAACTAACTCCAGTAATTCCTTGGTGGTACGAACATCCAATGAAGCACACAAGTTTGTTTGCTTTTCGTGCATGATATTAAATAGCTTGGCAGCAACAGGACTAGGATGAGTAGCAGCACGTTCCTTATATGTAGCTTTCGACATGATTTATCTTCGTTTCCTGCAGGTTTTTGTTCTGTGCAGTTGGGTTAAGAATACTGGGCAATTTCATGTTTCTTCAACACTACATATGCGTATATATACCAATCTAAGTCTGTGCTCCTTCCTTCGTTCTTCCTTCTGTTCGGAGATTACCGAATCAAAAAAATTTCAAAGAAACCGAAATCAAAAAAAAGAATAAAAAAAAAATGATGAATTGAATTGAAAAGCTGTGGTATGGTGCACTCTCAGTACAATCTGCTCTGATGCCGCATAGTTAAGCCAGCCCCGACACCCGCCAACACCCGCTGACGCGCCCTGACGGGCTTGTCTGCTCCCGGCATCCGCTTACAGACAAGCTGTGACCGTCTCCGGGAGCTGCATGTGTCAGAGGTTTTCACCGTCATCACCGAAACGCGCGA

> M249-5'clr-2-Ptub-clr-2-Bar-3'clr-2

TCGCGCGTTTCGGTGATGACGGTGAAAACCTCTGACACATGCAGCTCCCGGAGACGGTCACAGCTTGTCTGTAAGCGGATGCCGGGAGCAGACAAGCCCGTCAGGGCGCGTCAGCGGGTGTTGGCGGGTGTCGGGGCTGGCTTAACTATGCGGCATCAGAGCAGATTGTACTGAGAGTGCACCATACCACAGCTTTTCAATTCAATTCATCATTTTTTTTTTATTCTTTTTTTTGATTTCGGTTTCTTTGAAATTTTTTTGATTCGGTAATCTCCGAACAGAAGGAAGAACGAAGGAAGGAGCACAGACTTAGATTGGTATATATACGCATATGTAGTGTTGAAGAAACATGAAATTGCCCAGTATTCTTAACCCAACTGCACAGAACAAAAACCTGCAGGAAACGAAGATAAATCATGTCGAAAGCTACATATAAGGAACGTGCTGCTACTCATCCTAGTCCTGTTGCTGCCAAGCTATTTAATATCATGCACGAAAAGCAAACAAACTTGTGTGCTTCATTGGATGTTCGTACCACCAAGGAATTACTGGAGTTAGTTGAAGCATTAGGTCCCAAAATTTGTTTACTAAAAACACATGTGGATATCTTGACTGATTTTTCCATGGAGGGCACAGTTAAGCCGCTAAAGGCATTATCCGCCAAGTACAATTTTTTACTCTTCGAAGACAGAAAATTTGCTGACATTGGTAATACAGTCAAATTGCAGTACTCTGCGGGTGTATACAGAATAGCAGAATGGGCAGACATTACGAATGCACACGGTGTGGTGGGCCCAGGTATTGTTAGCGGTTTGAAGCAGGCGGCAGAAGAAGTAACAAAGGAACCTAGAGGCCTTTTGATGTTAGCAGAATTGTCATGCAAGGGCTCCCTATCTACTGGAGAATATACTAAGGGTACTGTTGACATTGCGAAGAGCGACAAAGATTTTGTTATCGGCTTTATTGCTCAAAGAGACATGGGTGGAAGAGATGAAGGTTACGATTGGTTGATTATGACACCCGGTGTGGGTTTAGATGACAAGGGAGACGCATTGGGTCAACAGTATAGAACCGTGGATGATGTGGTCTCTACAGGATCTGACATTATTATTGTTGGAAGAGGACTATTTGCAAAGGGAAGGGATGCTAAGGTAGAGGGTGAACGTTACAGAAAAGCAGGCTGGGAAGCATATTTGAGAAGATGCGGCCAGCAAAACTAAAAAACTGTATTATAAGTAAATGCATGTATACTAAACTCACAAATTAGAGCTTCAATTTAATTATATCAGTTATTACCCTATGCGGTGTGAAATACCGCACAGATGCGTAAGGAGAAAATACCGCATCAGGAAATTGTAAACGTTAATATTTTGTTAAAATTCGCGTTAAATTTTTGTTAAATCAGCTCATTTTTTAACCAATAGGCCGAAATCGGCAAAATCCCTTATAAATCAAAAGAATAGACCGAGATAGGGTTGAGTGTTGTTCCAGTTTGGAACAAGAGTCCACTATTAAAGAACGTGGACTCCAACGTCAAAGGGCGAAAAACCGTCTATCAGGGCGATGGCCCACTACGTGAACCATCACCCTAATCAAGTTTTTTGGGGTCGAGGTGCCGTAAAGCACTAAATCGGAACCCTAAAGGGAGCCCCCGATTTAGAGCTTGACGGGGAAAGCCGGCGAACGTGGCGAGAAAGGAAGGGAAGAAAGCGAAAGGAGCGGGCGCTAGGGCGCTGGCAAGTGTAGCGGTCACGCTGCGCGTAACCACCACACCCGCCGCGCTTAATGCGCCGCTACAGGGCGCGTCGCGCCATTCGCCATTCAGGCTGCGCAACTGTTGGGAAGGGCGATCGGTGCGGGCCTCTTCGCTATTACGCACACTTCTGGACGCGCTTTTCATCTTAATAATCTACGGATGTCGCTGTTTCTCATCTCTCGCTTGTCAATGTGCTTGGTTGTCAGCCCCCATGCGCGAGCCCTTTGAGAGTTCATAACATGATGACCTTCAAGTTGGGAAATAATCATGAGGCGACCAAGATGGACTCTTGCGAGATCAAATGTTGTGAGGAAGGCAAAGTTGCCTTTTATCATTGCTCAGTTATGGTCCAATGTTCTTGTCCCTACTTGCCTCAGAGCACAGTGTTGATGGACTTCGATTCCACATCTTTCCTAAAGGGCACAATTGGAACGAGAAGACTCACCTTGCTACAGTAACTTCAATTCTCAAAACCTAACTCGTCTAGAATATACTCGCGGATCTTCGACTTACCCAATGCTAGGCGACGTTCAAACATCAAACAGTCTGTTGAGCCGGTTCCTTCACAAAAGATTTCTATGTCAACACCTCTCCTCTCTTGTAACAAATCGAGCATTGCTTCCATTTGCCTTTATTGACGAACGACACCGGCGTGCCGACCTCCAGCAGTGATTGGCTAAATCCAGGCTGGAGGGGTTGACCAGCAACCCGAAACCTGTTGGAAGGATCTTTTACACTAGCTTGAGGAGGCTGTTACCACCGTTTTGTTAGCGCGTTCGCTCCGTCTTCTAGCTCCGTTCCCTTCACGATTCCTCTTCTAACTGCCAAGTGCCCACTCTCACTCTAGCTTCATCCACCCCGGCGAGCCCGGACCCCACCCCTGTCTGGCCCGATCGGACGGGAACTAGGGACAGAAATCGTAGCAAAGATCCACCATCATGCTGAGGTTCGCTGAAGACGGGGGGGAAGGAGAAGGAGGGGGGAGGGGGACTGGAGTGACGAACAGCTTTTCTTCCCCACAAGCGGCTGGCACCGAGGCAATAGCTACATACCTCTACCTACCTCCCTACGTATTTACCTTCATCATCTCAACCTCCAGTCAAATCGTCGAGTCAATATTGGCATATTCTGATTCCGTATATGTTGGGGAGCATTCCGTGCTTGTCCAGCTCCATCCTTCTTTTGTCTCTGTCCCCTCAACTTTGTTTACATGTACACCTGACTGACAAGAAGATGGGGAAATGAAGGTCATCATCCCTGCCATCATAGGCGACAGCGAAATGTTGCATGGCTGCTGCAATGCAGTGGACCTACATATTTCTAGTGTACTGCTCAATGTAAGGTACAAGTAACGAGCGATCCTTTCGTTATTTGGGTGAATGAGATCTCCTTTTTCCCAATCCCAACTTCCGGTGTTTTGTTCGATAAATGATATGCGACCAGGTTCAGGAGAGCGGGCTCATCCTTCCCTTCTGCGGGCAGTTGGCAATCAAGGTACACTACTACCCCAAATTGCCAATCAGCAGGCCCATACAGTACCCCGTACACTACCGTAAGCTACGAAGCTTGAAGCGTGCCGAGATGGAGGGGTAGCTTGCTTCCCTTGTTCCTTTGTAAGTGGAAGTCATGGATGGCATCAACTATACTAGTGTATAATGCCGCGTCGAATTGAGGAGTTTCCATCTATGTGTCTGTTTTTCCCTGATCTCCAGCTAGGTAATTTGCAGGTCGGGCTCAACAATGGATGTGAATGTAGCCCCCTACGAGTCGCGATGCTGTTCCTGATCTCCCAAGTCTGGCCGGGCCAGTGACCGAGTCGTGCCCAAAACCAACAGCCAAATCCAATTGGAGGTTGCGACAGTTCGACAGCGGTCTCGAGCTGGCGACTTACCCCACATTGGCAGGAGCATGTGGGACCCGCCGCCCGGCTGGTTTTGCCTGCCAACTCTGCCAGCCAACCAGAAGCAGACTAGCGCGTCGCGCTGGCCCCGAGAGACAGCTGGGGGAGGTTTGTCTTTGGCGGTTTTTGGAGGTCAACGCGTGAGGAACCGAGCCAGAGTGCAGGGACCACCCAAACCGAGTCGACAGGGGGCCTTCCACCCTTCCAAAAGTTTGACAGTGCCTGATGTCCCCACTTTGGCAAGCGCCCCGGCAACCCAGCAGCAGCCCGGCGAGCGGCGGCGGCGGTGAACGAGAAGCTGGTGCTGGCCGGCTGTGGCCCGCTCATTTCTTGCACCAAAAATCAACAAATCTCCCTTTCCACTCCCAAGACTTCCCAGTCTCCCCTAGGCCAGACATCAAACCTCTCCACTTTCACCGACCTCCGTCGTCTTCGTCTATCCGCGTCGGCTTTATCACCACTTCCATCTTTTTGCTCTTCCATCTTTCTGCCTTCAACCTCCACATAGACCCGTGTTCGTCATCACCAAACCGTCAAGATGGATTACAAGGATGATGATGATAAGGCACCATCAATGTCGATACCTACACAAGGCGGCATGTTTCACACATGTAAGTTTGATGACCTTTTCCAACCAAGGGGTCCATTACTAATGAGCAAATCCAGTCCAAGGAGTCACACCACGAAAGCCGGCTGTTGACTCTCGAGATAGTGTTAAGTCCAATGGTAGTGGGGCAGCCAAGAGGATAACTACTCCTCATGCTTGTGCTGAGTGTAAAAGACGAAAGATGTAAGTCAATGCGTGTCGATCAGCCTCACCATCAATCTAACTTGAGTGCCCTAGTCGGTGTGATGGTCAACAACCATGTGGCCAATGTCTCTCAAGCCGAGCACCGAAACGTTGTTTCTATGACAAGCACCGCCAAAGGGTGATCCCATCGCGCAAGACACTCGAAGCCCTCTCCCAGTCCCTCGAAGAATGCCGATCGATTTTGAAGCGACTGTATCCTCACCAAGAAGTCCACGCCTTGCTTCCCTTGTCCAGGCAAGAACTCCTCAACCTCCTGGATCGACCCGTCCCGGACAGCACAACAGCGAACGGACTACCGTCACCTCCTCTAAACACAACACCGATGGCCGAGTCAGAGACACCAACCAAGTCTGAAAACATTTTGGAACAGATTCCGACTCGGGATACAGAATGGGACGAGGAACGCCGGGAGAGGGACCATATCCCTGTAGAGGCGGACGATATCAACGCGCTGTCACTCTCGGTGGACAGACAAGCTTCTTATCTTGGAGCCTCATCCATCAAGGCTGCCCTTATGGTCATGCTCAAGGTTCAACCAGGCTTGAGGAGCTCCTTGGCCGCCCCGCTCAACAGTATCGAGATTTCTCATAACTTCCCGGCCATCAGACAGAAATCATCGAGTCAGAAAGATCCCCAGCGGATCCCATGGTCATGGAAGGGACAGACACTTATCGATGCCTACTTCAAGCGAGTCCACGTCTTCATCCCAATGCTCGACGAAACAACCTTCCGTGCCGATTACCTTGAAGGTCAACGATTTGACGCACCGTGGTTAGCCTTGTTGAACATGGTATTCGCCATGGGTAGCATTGTGGCAATGAAGTCGGACGATTACAATCACGTCAACTATTACAACCGCGCCATGGAGCATCTTCCCATGGATTCCTTTGGCAGCAGCCATATCGAGACTGTCCAAGCATTGGCACTCATCGGTGGTTACTATCTTCACTATATTAACCGACCCAACATGGCCAACGCTGTTTTGGGTGCCGCCATCCGCATGGCCAGCGCTCTCGGTCTCCACCGTGAGAGTCTTGCCCAAGGAGGAAGTGATATGGTCGCTGCCGAGACCAGGAGACGCACATGGTGGGCTCTTTTCTGCCTTGACACGTGGGCCACGACAACCATGGGACGTCCCTCTTTTGGCCGTTGGGGACCCGCCATCAACATTCGGCCGCCCGAGTTCGGTGTGAATGCCGGTCGGGACTCGTCTCAACACGCGGGTATTCTCCCCATGATTGAAAACGTCAAGTTCTGCAAAATCGCCACTCAGATCCAAGACATGCTCGCCATTTCTCCTTTGCTGCGTACCGAGGACCGCTGCAATCTGGACGGACAGCTCGTTGTCTGGTATGAAAACCTACCTTGGCTCCTGCGTACGACCGACCCATGCGCTGAGCCGCTCTACATGGCCCGCTGCATCATGAAGTGGCGGTATCAGAACCTCCGTATGCTTCTTCATCGCCCAGTCCTCCTCTCCATGGCCTCATCAGGTCTTAACCCACATACTCAGGCCTGTGACGCGGATCTCGCCGCCATTGAGACGTGCCGAGAGCTTGCTGCGGCAACCATTGAGGACATTGGTCGTGAATGGACACGTAACCAGATGTCCGGCTGGAACGCAGTGTGGTTTCTCTACCAGGCTGCCATGGTTCCTCTTGTCTCCGTCTTTTGGCAATGGGGCAACCCTCGTGTCCCTGAGTGGCTCAAGCAGATTGAGGCGGTATTGGAGCTCCTTGAGGCCATGGAAGAGTGGTCACTCGCTGCTCGCAGGAGCCGGGAGGTGGTTCTTCGCATGTACGAGGCCAGCAGAGTCATCCAGGCACAGGGTGCCGCAGCGCACCAACTACAGCAGAGGGGATCGCAGAGCCCTCATTCTCTCAGCAGCACTACTGCGTCCTTGGGCAACATGCACATCAACAACGACTTGCTCATGGGGTCGTCACCGGGTTCGGCGGAGCTCTACATGACTCCTATCGACCTGGAGCCCGTCGAAGGCTTGGGCATGACGGGTGTGCTGGACCACGGCGGGATGTGGGATCTCGACGGCATGCTTTGGGGTCCCAGTCCAAGCCCGTCGGTTCACCATGGTCAACACTCGACTCACCCGGATGATGCGGTGCCCACGGTGGCGGAGTACGCTGCAGCGTTCCCGACGGCTGATGTAGTAGATGGGTTTCTGCAACATCACTCGGCTATGGAGTTTGGGAACATGATGGGTCACCACCACCACCACCACCATGCAGGTGCACATGCTGGCCAGGGTCAGTTTGGAGGTATGGATGGGTACACCTACTAGTTCTCGGTACTATGCATTACATGTTAATTATTACATGCTTAACGTAATTCAACAGAAATTATATGATAATCATCGCAAGACCGGCAACAGGATTCAATCTTAAGAAACTTTATTGCCAAATGTTTGAACGATCTGCAGGTCGACGGATCAGATCTCGGTGACGGGCAGGACCGGACGGGGCGGTACCGGCAGGCTGAAGTCCAGCTGCCAGAAACCCACGTCATGCCAGTTCCCGTGCTTGAAGCCGGCCGCCCGCAGCATGCCGCGGGGGGCATATCCGAGCGCCTCGTGCATGCGCACGCTCGGGTCGTTGGGCAGCCCGATGACAGCGACCACGCTCTTGAAGCCCTGTGCCTCCAGGGACTTCAGCAGGTGGGTGTAGAGCGTGGAGCCCAGTCCCGTCCGCTGGTGGCGGGGGGAGACGTACACGGTCGACTCGGCCGTCCAGTCGTAGGCGTTGCGTGCCTTCCAGGGGCCCGCGTAGGCGATGCCGGCGACCTCGCCGTCCACCTCGGCGACGAGCCAGGGATAGCGCTCCCGCAGACGGACGAGGTCGTCCGTCCACTCCTGCGGTTCCTGCGGCTCGGTACGGAAGTTGACCGTGCTTGTCTCGATGTAGTGGTTGACGATGGTGCAGACCGCCGGCATGTCCGCCTCGGTGGCACGGCGGATGTCGGCCGGGCGTCGTTCTGGGCTCATGGTTACTTCCTAATCGAAGCTTTGCTTGGGTAGAATAGGTAAGTCAGATTGAATCTGAAATAAAGGGAGGAAGGGCGAACTTAAGAAGGTATGACCGGGTCGTTCACTTACCTTGCTTGACAAACGCACCAAGTTATCGTGCACCAAGCAGCAGATGATAATAATGTCCTCGTTCCTGTCTGCTAATAAGAGTCACACTTCGAGCGCCGCCGCTACTGCTACAAGTGGGGCTGATCTGACCAGTTGCCTAAATGAACCATCTTGTCAAACGACACAAATTTTGTGCTCACCGCCTGGACGACTAAACCAAAATAGGCATTCATTGTTGACCTCCACTAGCTCCAGCCAAGCCCAAAAAGTGCTCCTTCAATATCATCTTCTGTCGAGTCTAGAAGGCCCCTCGCAGTGCTCCAATTTTCTAGTTTACTACTCATTTCTCTCTTTCTTTCTTTCTTTCTTCCTCTTTTCACACAAAAAATAGTTGGGCTGGAAGCCCGGGGTTATGCACAGTTGGCGCGACGCAGGCTTGAAGGACCGAAATACCACCACATACGATGGCTGGATGCGGGTGGTGCGGATTTGGCTTACGAATGGTTGCTTTTTGCGGGGTACATCATATACGGAGAAGGATAAGGGTCTCATGGTAAAAAGGAAAAGGGAGCTTTGTTTTTTGGCGGTTGATTTCTCAGGGAATGTTGGTTTGATTTTTGGCTGATTTTGGATGGTTACAACGGCGCCTTCGCGGGTACACTCTCCCTCTTGTTAGGTGTAGTGTCCCACCTCCCTTCCATTCACCAGCCACCGGCCTTACCTAGGCCGTTGTCATGTGGGCCTAGTTCAGGGCACCACCATGTTCATTGTATTATTATTATAGCCCTTTTTACATTTCATTTCCACTTTAGCTTCTTAACACAGTCATCAACACTTCTTGTGCCTACACTGCGGCATCTCTTTTCGCCATCGCTTGCAGTACACTAGTTCTGATGTCACATGCGGTTATGTTTTTATCCCGTTGTACCACTTTGTGCCTCTTGGTCCATTGACGAGGCCTTCCTCTCCTTCTTCGATCTTTGAGCCTTTGTCCGATAAGGGGCGGTTATGAGAAAATGGAAGAATGGAAGAAAAATCTTATGATAAAGAAACACAACATAGAAACTTGGCTTTAACAACTCTTGTTCATATGCTGTACACATGTAGTTACCGACCTGCTTGGCCATGTCGTTGAAGTTGTCTGGTGTCACATAAACCATCAAGAACCATAATGCAGCAGCAGATTGCATTTCATGCTCCACAAAGAGAAAGGGTCCTCAGCTGTACCTGGGATAGATAGTTGGATGTACGGTGCAAGACATGGCTAGTACGTACTTACCTAAAGGTAGGTTTAGGTTGCATACTTTTGACTTTAGGCGAGAGTGAAAGTGGTACATTAATGAATCGGCCAACGCGCGGGGAGAGGCGGTTTGCGTATTGGGCGCTCTTCCGCTTCCTCGCTCACTGACTCGCTGCGCTCGGTCGTTCGGCTGCGGCGAGCGGTATCAGCTCACTCAAAGGCGGTAATACGGTTATCCACAGAATCAGGGGATAACGCAGGAAAGAACATGTGAGCAAAAGGCCAGCAAAAGGCCAGGAACCGTAAAAAGGCCGCGTTGCTGGCGTTTTTCCATAGGCTCCGCCCCCCTGACGAGCATCACAAAAATCGACGCTCAAGTCAGAGGTGGCGAAACCCGACAGGACTATAAAGATACCAGGCGTTTCCCCCTGGAAGCTCCCTCGTGCGCTCTCCTGTTCCGACCCTGCCGCTTACCGGATACCTGTCCGCCTTTCTCCCTTCGGGAAGCGTGGCGCTTTCTCATAGCTCACGCTGTAGGTATCTCAGTTCGGTGTAGGTCGTTCGCTCCAAGCTGGGCTGTGTGCACGAACCCCCCGTTCAGCCCGACCGCTGCGCCTTATCCGGTAACTATCGTCTTGAGTCCAACCCGGTAAGACACGACTTATCGCCACTGGCAGCAGCCACTGGTAACAGGATTAGCAGAGCGAGGTATGTAGGCGGTGCTACAGAGTTCTTGAAGTGGTGGCCTAACTACGGCTACACTAGAAGGACAGTATTTGGTATCTGCGCTCTGCTGAAGCCAGTTACCTTCGGAAAAAGAGTTGGTAGCTCTTGATCCGGCAAACAAACCACCGCTGGTAGCGGTGGTTTTTTTGTTTGCAAGCAGCAGATTACGCGCAGAAAAAAAGGATCTCAAGAAGATCCTTTGATCTTTTCTACGGGGTCTGACGCTCAGTGGAACGAAAACTCACGTTAAGGGATTTTGGTCATGAGATTATCAAAAAGGATCTTCACCTAGATCCTTTTAAATTAAAAATGAAGTTTTAAATCAATCTAAAGTATATATGAGTAAACTTGGTCTGACAGTTACCAATGCTTAATCAGTGAGGCACCTATCTCAGCGATCTGTCTATTTCGTTCATCCATAGTTGCCTGACTCCCCGTCGTGTAGATAACTACGATACGGGAGGGCTTACCATCTGGCCCCAGTGCTGCAATGATACCGCGAGACCCACGCTCACCGGCTCCAGATTTATCAGCAATAAACCAGCCAGCCGGAAGGGCCGAGCGCAGAAGTGGTCCTGCAACTTTATCCGCCTCCATCCAGTCTATTAATTGTTGCCGGGAAGCTAGAGTAAGTAGTTCGCCAGTTAATAGTTTGCGCAACGTTGTTGCCATTGCTACAGGCATCGTGGTGTCACGCTCGTCGTTTGGTATGGCTTCATTCAGCTCCGGTTCCCAACGATCAAGGCGAGTTACATGATCCCCCATGTTGTGCAAAAAAGCGGTTAGCTCCTTCGGTCCTCCGATCGTTGTCAGAAGTAAGTTGGCCGCAGTGTTATCACTCATGGTTATGGCAGCACTGCATAATTCTCTTACTGTCATGCCATCCGTAAGATGCTTTTCTGTGACTGGTGAGTACTCAACCAAGTCATTCTGAGAATAGTGTATGCGGCGACCGAGTTGCTCTTGCCCGGCGTCAATACGGGATAATACCGCGCCACATAGCAGAACTTTAAAAGTGCTCATCATTGGAAAACGTTCTTCGGGGCGAAAACTCTCAAGGATCTTACCGCTGTTGAGATCCAGTTCGATGTAACCCACTCGTGCACCCAACTGATCTTCAGCATCTTTTACTTTCACCAGCGTTTCTGGGTGAGCAAAAACAGGAAGGCAAAATGCCGCAAAAAAGGGAATAAGGGCGACACGGAAATGTTGAATACTCATACTCTTCCTTTTTCAATATTATTGAAGCATTTATCAGGGTTATTGTCTCATGAGCGGATACATATTTGAATGTATTTAGAAAAATAAACAAATAGGGGTTCCGCGCACATTTCCCCGAAAAGTGCCACCTGAACGAAGCATCTGTGCTTCATTTTGTAGAACAAAAATGCAACGCGAGAGCGCTAATTTTTCAAACAAAGAATCTGAGCTGCATTTTTACAGAACAGAAATGCAACGCGAAAGCGCTATTTTACCAACGAAGAATCTGTGCTTCATTTTTGTAAAACAAAAATGCAACGCGAGAGCGCTAATTTTTCAAACAAAGAATCTGAGCTGCATTTTTACAGAACAGAAATGCAACGCGAGAGCGCTATTTTACCAACAAAGAATCTATACTTCTTTTTTGTTCTACAAAAATGCATCCCGAGAGCGCTATTTTTCTAACAAAGCATCTTAGATTACTTTTTTTCTCCTTTGTGCGCTCTATAATGCAGTCTCTTGATAACTTTTTGCACTGTAGGTCCGTTAAGGTTAGAAGAAGGCTACTTTGGTGTCTATTTTCTCTTCCATAAAAAAAGCCTGACTCCACTTCCCGCGTTTACTGATTACTAGCGAAGCTGCGGGTGCATTTTTTCAAGATAAAGGCATCCCCGATTATATTCTATACCGATGTGGATTGCGCATACTTTGTGAACAGAAAGTGATAGCGTTGATGATTCTTCATTGGTCAGAAAATTATGAACGGTTTCTTCTATTTTGTCTCTATATACTACGTATAGGAAATGTTTACATTTTCGTATTGTTTTCGATTCACTCTATGAATAGTTCTTACTACAATTTTTTTGTCTAAAGAGTAATACTAGAGATAAACATAAAAAATGTAGAGGTCGAGTTTAGATGCAAGTTCAAGGAGCGAAAGGTGGATGGGTAGGTTATATAGGGATATAGCACAGAGATATATAGCAAAGAGATACTTTTGAGCAATGTTTGTGGAAGCGGTATTCGCAATATTTTAGTAGCTCGTTACAGTCCGGTGCGTTTTTGGTTTTTTGAAAGTGCGTCTTCAGAGCGCTTTTGGTTTTCAAAAGCGCTCTGAAGTTCCTATACTTTCTAGAGAATAGGAACTTCGGAATAGGAACTTCAAAGCGTTTCCGAAAACGAGCGCTTCCGAAAATGCAACGCGAGCTGCGCACATACAGCTCACTGTTCACGTCGCACCTATATCTGCGTGTTGCCTGTATATATATATACATGAGAAGAACGGCATAGTGCGTGTTTATGCTTAAATGCGTACTTATATGCGTCTATTTATGTAGGATGAAAGGTAGTCTAGTACCTCCTGTGATATTATCCCATTCCATGCGGGGTATCGTATGCTTCCTTCAGCACTACCCTTTAGCTGTTCTATATGCTGCCACTCCTCAATTGGATTAGTCTCATCCTTCAATGCTATCATTTCCTTTGATATTGGATCATACTAAGAAACCATTATTATCATGACATTAACCTATAAAAATAGGCGTATCACGAGGCCCTTTCGTC

> M249-5'csr-1-Pgsy-1-luciferase-Bar-3'csr-1

TCGCGCGTTTCGGTGATGACGGTGAAAACCTCTGACACATGCAGCTCCCGGAGACGGTCACAGCTTGTCTGTAAGCGGATGCCGGGAGCAGACAAGCCCGTCAGGGCGCGTCAGCGGGTGTTGGCGGGTGTCGGGGCTGGCTTAACTATGCGGCATCAGAGCAGATTGTACTGAGAGTGCACCATACCACAGCTTTTCAATTCAATTCATCATTTTTTTTTTATTCTTTTTTTTGATTTCGGTTTCTTTGAAATTTTTTTGATTCGGTAATCTCCGAACAGAAGGAAGAACGAAGGAAGGAGCACAGACTTAGATTGGTATATATACGCATATGTAGTGTTGAAGAAACATGAAATTGCCCAGTATTCTTAACCCAACTGCACAGAACAAAAACCTGCAGGAAACGAAGATAAATCTAGATCCAATCTGAGCCTCGCGGTGATGATTGCCAGACGGGCGGGATGCTGTGGCGACTTGGTGGTTTTTGTTCGGGCTGATCGCTTTCAGGGCGACGTTTCACGGTCTTGTCGGGATCAGATGGACATTGAGGTGGCCTAGCGTCAGGGTGCCGCTGAGCGCTGACCTCAACTGACGCCGTCCAAGGCCGACCCCTGCGCTTGACCCAACACCCTGACACCAAGAAGCCAGTGGAACCCAGGAGGTCTGCCAAGACTTTGAAAGCTTGGCACGAGCGTCTCAGCTCTTTGAAGCCTGAGTTTTCAGGCAAGAATTCTGCTTGTAATTGCCGGGCCGGCCGACAACGCGGGGCGGCGGGAGGGGACGCTCGGCCGCCGGAGCTCGCTTCCATGCATTTTGGATGGAGTAAAAATTGTTAAACACCCGACCCGACTCCGTTCTCCCTCTCGTCTCAGGTCTTTGTTGTGCATCCATCGCCGTGTAGCAGATTTCCATCGTTCGTTTGTTCGGACTTTACCTTGTACTTTTAGCTTCATTATCATCCTACAATCATCCAGGATTTCATCTTCCACAACCGGTACGTTATCATTTTTACTTTTGTTGCCATGTTCTTCTTGAGCCTTTTCTGTCTAGAGGAGCTCTCCCTGCCGGCTGGGAGCTTGCCCAAAGCTCCAAGCTCTATCTGTGGCAGAGCTCTGAGAGCTTCCATTCCCGGGATTTCTTGGCATCAGATTCAATTCGAATGTCCTTTTTTCCATCCACGCTCGAGATGAGGGGTGAGGTTTGTTAGTTTGACAATACCACCACCGCCTTTTGGTCCCTACCCCAGACATGGCCATCAAGGTCGTTAATGCCGTCCATCCCTCTTGTTCGGCTTGGGGTATCGTCCATGGCTCCGCAATGTCGTCAATGGCTGCATTGACAGAGGGGGACTCCAAATGAAAGAGGCAATCGCAACGCTATGAAGACCCAACAAGCCATATGATGGAGCCATGGGTCTACGGTCCTTTGGCCCGGATGTCTTTGGATAGCTCTTGCCAGCGCTTTAGCTGCCAACTATACACACACCGGAATTATACGATTTAGGTGACTGCAGGCGAGATCGAGCAACCCAGAAAGAAGGGGAACCTTTTGGCGGCGGCAACCTGGAATGCATAGCATTGGATCCCATTGAGTTAGGTTTGCAATATCATGATCTGTACGTAGGAAGAAGGTATCTGTGTAGCAGTGTTGCCCCCCAGTTCAAGAGTAGCCGAGTATGTATAAACAAACCTCAAACTTCTCCCCTCTTCTATAGCGTTACTGCCCAATTACCTACCTCTACCTATCCTATCCAAAGAAACCGGCGGCACTACCTACCTACCACTACCGACTTACCCTCTCGCTCCCGCTTTCAGACTTCCTTCAGTCATCCTGTTTCAGCTTCACTTCAGACTGTCAACGTAGCGTAGGTAGTCTTTTGTCTGTCCTACCGTGCCCTTCCCGTTCGCAGCCGACTTCATCTCGCAGCGAGCCCACTTACACGCGCCTCCCCTTGTTCACGAGGACCTCGCGTTACTCAGATTCTGACTCTTCGGGTTGGGTCTAGTATAGTGAGATTCAGTTCCAGATCTGCCTCTGTTGCTGCCCGAAGGCACTGTTTTCCAGTTCCGTTTGTGCCTCCCTGCCCTAGAGGTGGAACTCTTCGAGGTACGTTCCGGTTTGTGAGACACACACTTTTTCTCTTCCTGCGACACCGCAGCACCTGTGTCGAGCCGTCGAGCCGGTGGTATTCGCAGCCCAGACTCCCAAGCGAACCTAGCTCAAGAGATCGAAGCCGTGCGCCGGGAAGAGGTTGGAAACTCGGTTCAGGTTGAGGTTCCAGTAGCTTCAGTGAGGCCCCGTTCCCCGCTTCCGGCCGGGCTTTTAAAGCTTCGCATCCCCAGCTGCACCCCTGAAGCTTCCCACCCAGACCCCATTCCGGGTGACATTCCTCGAAGCTCTACCCCACCAGTGCGTGTTGTCTCGTCCTGGCTACGTCGACCTGATCTGCTTCAAATAGAACCCAACCGTCTGCTGGTCCCTCTTCTTGCTACGTCTACTCGCCCTCTCTTCCTCTCTACGAACCTGAAAAATCAGTGTTCTGTGGCAGCTTCTGGTTACCTACTAACACGAAGCTCGGACGACAGAAGCATCTTGACTTCGGCGCGCCCGTCACCCATTCATATTGAGCCACAATGGAGGACGCCAAGAACATCAAGAAGGGCCCCGCCCCCTTCTACCCCCTCGAGGACGGCACCGCCGGCGAGCAGCTCCACAAGGCCATGAAGCGCTACGCCCTCGTCCCCGGCACCATCGCCTTCGTAGGTTTCCTCCAGCTCTCGCCTCCAGCACCCGAGGCACATCTCGGGCATCTTCACAACAACAGACACTGACATCTCATTCTCACAGACCGACGCCCACATCGAGGTCAACATCACCTACGCCGAGTACTTCGAGATGTCCGTCCGCCTCGCCGAGGCCATGAAGCGCTACGGCCTCAACACCAACCACCGCATCGTCGTCTGCTCCGAGAACTCCCTCCAGTTCTTCATGCCCGTCCTCGGCGCCCTCTTCATCGGCGTCGCCGTCGCCCCCGCCAACGACATCTACAACGAGCGCGAGCTCCTCAACTCCATGAACATCTCCCAGCCCACCGTCGTCTTCGTCTCCAAGAAGGGCCTCCAGAAGATCCTCAACGTCCAGAAGAAGCTCCCCATCATCCAGAAGATCATCATCATGGACTCCAAGACCGACTACCAGGGCTTCCAGTCCATGTACACCTTCGTCACCTCCCACCTCCCCCCCGGCTTCAACGAGTACGACTTCGTCCCCGAGTCCTTCGACCGCGACAAGACCATCGCCCTCATCATGAACTCCTCCGGCTCCACCGGCCTCCCCAAGGGCGTCGCCCTCCCCCACCGCACCGCCTGCGTCCGCTTCTCCCACGCCCGCGACCCCATCTTCGGCAACCAGATCATCCCCGACACCGCCATCCTCTCCGTCGTCCCCTTCCACCACGGCTTCGGCATGTTCACCACCCTCGGCTACCTCATCTGCGGCTTCCGCGTCGTCCTCATGTACCGCTTCGAGGAGGAGCTCTTCCTCCGCTCCCTCCAGGACTACAAGATCCAGTCCGCCCTCCTCGTCCCCACCCTCTTCTCCTTCTTCGCCAAGTCCACCCTCATCGACAAGTACGACCTCTCCAACCTCCACGAGATCGCCTCCGGCGGCGCCCCCCTCTCCAAGGAGGTCGGCGAGGCCGTCGCCAAGCGCTTCCACCTCCCCGGCATCCGCCAGGGCTACGGCCTCACCGAGACCACCTCCGCCATCCTCATCACCCCCGAGGGCGACGACAAGCCCGGCGCCGTCGGCAAGGTCGTCCCCTTCTTCGAGGCCAAGGTCGTCGACCTCGACACCGGCAAGACCCTCGGCGTCAACCAGCGCGGCGAGCTCTGCGTCCGCGGCCCCATGATCATGTCCGGCTACGTCAACAACCCCGAGGCCACCAACGCCCTCATCGACAAGGACGGCTGGCTCCACTCCGGCGACATCGCCTACTGGGACGAGGACGAGCACTTCTTCATCGTCGACCGCCTCAAGTCCCTCATCAAGTACAAGGGCTACCAGGTCGCCCCCGCCGAGCTCGAGTCCATCCTCCTCCAGCACCCCAACATCTTCGACGCCGGCGTCGCCGGCCTCCCCGACGACGACGCCGGCGAGCTCCCCGCCGCCGTCGTCGTCCTCGAGCACGGCAAGACCATGACCGAGAAGGAGATCGTCGACTACGTCGCCTCCCAGGTCACCACCGCCAAGAAGCTCCGCGGCGGCGTCGTCTTCGTCGACGAGGTCCCCAAGGGCCTCACCGGCAAGCTCGACGCCCGCAAGATCCGCGAGATCCTCATCAAGGCCAAGAAGGGCGGCAAGTCCAAGCTCCTAGAGGATCCGACACTATAGAATACCTATTCTCGGTACTATGCATTACATGTTAATTATTACATGCTTAACGTAATTCAACAGAAATTATATGATAATCATCGCAAGACCGGCAACAGGATTCAATCTTAAGAAACTTTATTGCCAAATGTTTGAACGATCTGCAGGTCGACGGATCAGATCTCGGTGACGGGCAGGACCGGACGGGGCGGTACCGGCAGGCTGAAGTCCAGCTGCCAGAAACCCACGTCATGCCAGTTCCCGTGCTTGAAGCCGGCCGCCCGCAGCATGCCGCGGGGGGCATATCCGAGCGCCTCGTGCATGCGCACGCTCGGGTCGTTGGGCAGCCCGATGACAGCGACCACGCTCTTGAAGCCCTGTGCCTCCAGGGACTTCAGCAGGTGGGTGTAGAGCGTGGAGCCCAGTCCCGTCCGCTGGTGGCGGGGGGAGACGTACACGGTCGACTCGGCCGTCCAGTCGTAGGCGTTGCGTGCCTTCCAGGGGCCCGCGTAGGCGATGCCGGCGACCTCGCCGTCCACCTCGGCGACGAGCCAGGGATAGCGCTCCCGCAGACGGACGAGGTCGTCCGTCCACTCCTGCGGTTCCTGCGGCTCGGTACGGAAGTTGACCGTGCTTGTCTCGATGTAGTGGTTGACGATGGTGCAGACCGCCGGCATGTCCGCCTCGGTGGCACGGCGGATGTCGGCCGGGCGTCGTTCTGGGCTCATGGTTACTTCCTAATCGAAGCTTTGCTTGGGTAGAATAGGTAAGTCAGATTGAATCTGAAATAAAGGGAGGAAGGGCGAACTTAAGAAGGTATGACCGGGTCGTTCACTTACCTTGCTTGACAAACGCACCAAGTTATCGTGCACCAAGCAGCAGATGATAATAATGTCCTCGTTCCTGTCTGCTAATAAGAGTCACACTTCGAGCGCCGCCGCTACTGCTACAAGTGGGGCTGATCTGACCAGTTGCCTAAATGAACCATCTTGTCAAACGACACAAATTTTGTGCTCACCGCCTGGACGACTAAACCAAAATAGGCATTCATTGTTGACCTCCACTAGCTCCAGCCAAGCCCAAAAAGTGCTCCTTCAATATCATCTTCTGTCGAGTCTAGATGACTTGTTCCTGTACCTCGTCACCATCCAGCCACCGTCTGAAATAGACGCAGTTGCTGGGGCTGACAGGTCCGCCTTCCTCGCACATATCATAGACAGGGCACCGCCCACAAGGCGCCTCCATAAGGCCACTTGCACTCGGCTCAACTCCCATAAATGAGGTAACAATGCCTCCTTTGGTTGGATCATGGTCGAGGCGTCGTTGATTGCACGTCCTCGGATCCTGTTTGGGGATGCGCGTCACACGGTACCCCTTGCGCCCATTGACCTTGACAGGCTCTACTAAGCCATCGTAGTATAGCACATCCACCAACTGCTGGATATCCGCCTCGCTCAACGTCGTATTATTCGTGATGCCGGCGTGGTGGATGAACTTTGCAATGTCCGACACAGTCGGGTACGACTTGTAACCGGCCGGGAGAGGCAGCAATTGCGGCTTGCCGGGCCTGGTCTTCTTGGCCGGTGCCGGTGCCGCCGTGTCGTCGTTGGAAATCTCGCCCGCGCTGCGCTTCGTCCCCTTGGAAATATCCTCCGAGTGGGGAATGGTCCCCTGAACCACGCCCTTCTTGGGATTCTTTTCACGCTGCTGTGCTCCTCCTCCTTGTGCTCCTACTCCTGGTACTCCAGCACCGCCGCCGTGGGAAGTATATGTCGATTTCTGCTTGATAAAGTCAAACACAATTTCCTGTAGCTCGTTGATGAAGGCCGTGTCGAGCTCGCCCTCAGTAAACCAGGGGCCACCTGTGGCGCGATCGGAGGGACGTAGGTTCGCCTTGATGTACATCTTCTTGTTGGGGTGCTCGACGTTCTTCATGCTGGCGATGTAGCCCTTTTGCTCGAGATACTTGATGCAATGCTTCATAAGCGAGTCGGCCATGTTGAGCTTGTTCTTGATGGTCCGGTTCCAGATGCCGTCGGCCCCGGCTTCGTCGACCATGGCGTATACCATCATTATTTCGGAGTTGGGGAGGGAGGTGTACCTTTTGGGAGGAAGGGGAGGTTAGTTGGTGTGCTCATTTAAATGCTGTTTCCTGTGTGAAATTGTTATCCGCTCACAATTCCACACAACATAGGAGCCGGAAGCATAAAGTGTAAAGCCTGGGGTGCCTAATGAGTGAGGTAACTCACATTAATTGCGTTGCGCTCACTGCCCGCTTTCCAGTCGGGAAACCTGTCGTGCCAGCTGCATTAATGAATCGGCCAACGCGCGGGGAGAGGCGGTTTGCGTATTGGGCGCTCTTCCGCTTCCTCGCTCACTGACTCGCTGCGCTCGGTCGTTCGGCTGCGGCGAGCGGTATCAGCTCACTCAAAGGCGGTAATACGGTTATCCACAGAATCAGGGGATAACGCAGGAAAGAACATGTGAGCAAAAGGCCAGCAAAAGGCCAGGAACCGTAAAAAGGCCGCGTTGCTGGCGTTTTTCCATAGGCTCCGCCCCCCTGACGAGCATCACAAAAATCGACGCTCAAGTCAGAGGTGGCGAAACCCGACAGGACTATAAAGATACCAGGCGTTTCCCCCTGGAAGCTCCCTCGTGCGCTCTCCTGTTCCGACCCTGCCGCTTACCGGATACCTGTCCGCCTTTCTCCCTTCGGGAAGCGTGGCGCTTTCTCATAGCTCACGCTGTAGGTATCTCAGTTCGGTGTAGGTCGTTCGCTCCAAGCTGGGCTGTGTGCACGAACCCCCCGTTCAGCCCGACCGCTGCGCCTTATCCGGTAACTATCGTCTTGAGTCCAACCCGGTAAGACACGACTTATCGCCACTGGCAGCAGCCACTGGTAACAGGATTAGCAGAGCGAGGTATGTAGGCGGTGCTACAGAGTTCTTGAAGTGGTGGCCTAACTACGGCTACACTAGAAGGACAGTATTTGGTATCTGCGCTCTGCTGAAGCCAGTTACCTTCGGAAAAAGAGTTGGTAGCTCTTGATCCGGCAAACAAACCACCGCTGGTAGCGGTGGTTTTTTTGTTTGCAAGCAGCAGATTACGCGCAGAAAAAAAGGATCTCAAGAAGATCCTTTGATCTTTTCTACGGGGTCTGACGCTCAGTGGAACGAAAACTCACGTTAAGGGATTTTGGTCATGAGATTATCAAAAAGGATCTTCACCTAGATCCTTTTAAATTAAAAATGAAGTTTTAAATCAATCTAAAGTATATATGAGTAAACTTGGTCTGACAGTTACCAATGCTTAATCAGTGAGGCACCTATCTCAGCGATCTGTCTATTTCGTTCATCCATAGTTGCCTGACTCCCCGTCGTGTAGATAACTACGATACGGGAGGGCTTACCATCTGGCCCCAGTGCTGCAATGATACCGCGAGACCCACGCTCACCGGCTCCAGATTTATCAGCAATAAACCAGCCAGCCGGAAGGGCCGAGCGCAGAAGTGGTCCTGCAACTTTATCCGCCTCCATCCAGTCTATTAATTGTTGCCGGGAAGCTAGAGTAAGTAGTTCGCCAGTTAATAGTTTGCGCAACGTTGTTGCCATTGCTACAGGCATCGTGGTGTCACGCTCGTCGTTTGGTATGGCTTCATTCAGCTCCGGTTCCCAACGATCAAGGCGAGTTACATGATCCCCCATGTTGTGCAAAAAAGCGGTTAGCTCCTTCGGTCCTCCGATCGTTGTCAGAAGTAAGTTGGCCGCAGTGTTATCACTCATGGTTATGGCAGCACTGCATAATTCTCTTACTGTCATGCCATCCGTAAGATGCTTTTCTGTGACTGGTGAGTACTCAACCAAGTCATTCTGAGAATAGTGTATGCGGCGACCGAGTTGCTCTTGCCCGGCGTCAATACGGGATAATACCGCGCCACATAGCAGAACTTTAAAAGTGCTCATCATTGGAAAACGTTCTTCGGGGCGAAAACTCTCAAGGATCTTACCGCTGTTGAGATCCAGTTCGATGTAACCCACTCGTGCACCCAACTGATCTTCAGCATCTTTTACTTTCACCAGCGTTTCTGGGTGAGCAAAAACAGGAAGGCAAAATGCCGCAAAAAAGGGAATAAGGGCGACACGGAAATGTTGAATACTCATACTCTTCCTTTTTCAATATTATTGAAGCATTTATCAGGGTTATTGTCTCATGAGCGGATACATATTTGAATGTATTTAGAAAAATAAACAAATAGGGGTTCCGCGCACATTTCCCCGAAAAGTGCCACCTGAACGAAGCATCTGTGCTTCATTTTGTAGAACAAAAATGCAACGCGAGAGCGCTAATTTTTCAAACAAAGAATCTGAGCTGCATTTTTACAGAACAGAAATGCAACGCGAAAGCGCTATTTTACCAACGAAGAATCTGTGCTTCATTTTTGTAAAACAAAAATGCAACGCGAGAGCGCTAATTTTTCAAACAAAGAATCTGAGCTGCATTTTTACAGAACAGAAATGCAACGCGAGAGCGCTATTTTACCAACAAAGAATCTATACTTCTTTTTTGTTCTACAAAAATGCATCCCGAGAGCGCTATTTTTCTAACAAAGCATCTTAGATTACTTTTTTTCTCCTTTGTGCGCTCTATAATGCAGTCTCTTGATAACTTTTTGCACTGTAGGTCCGTTAAGGTTAGAAGAAGGCTACTTTGGTGTCTATTTTCTCTTCCATAAAAAAAGCCTGACTCCACTTCCCGCGTTTACTGATTACTAGCGAAGCTGCGGGTGCATTTTTTCAAGATAAAGGCATCCCCGATTATATTCTATACCGATGTGGATTGCGCATACTTTGTGAACAGAAAGTGATAGCGTTGATGATTCTTCATTGGTCAGAAAATTATGAACGGTTTCTTCTATTTTGTCTCTATATACTACGTATAGGAAATGTTTACATTTTCGTATTGTTTTCGATTCACTCTATGAATAGTTCTTACTACAATTTTTTTGTCTAAAGAGTAATACTAGAGATAAACATAAAAAATGTAGAGGTCGAGTTTAGATGCAAGTTCAAGGAGCGAAAGGTGGATGGGTAGGTTATATAGGGATATAGCACAGAGATATATAGCAAAGAGATACTTTTGAGCAATGTTTGTGGAAGCGGTATTCGCAATATTTTAGTAGCTCGTTACAGTCCGGTGCGTTTTTGGTTTTTTGAAAGTGCGTCTTCAGAGCGCTTTTGGTTTTCAAAAGCGCTCTGAAGTTCCTATACTTTCTAGAGAATAGGAACTTCGGAATAGGAACTTCAAAGCGTTTCCGAAAACGAGCGCTTCCGAAAATGCAACGCGAGCTGCGCACATACAGCTCACTGTTCACGTCGCACCTATATCTGCGTGTTGCCTGTATATATATATACATGAGAAGAACGGCATAGTGCGTGTTTATGCTTAAATGCGTACTTATATGCGTCTATTTATGTAGGATGAAAGGTAGTCTAGTACCTCCTGTGATATTATCCCATTCCATGCGGGGTATCGTATGCTTCCTTCAGCACTACCCTTTAGCTGTTCTATATGCTGCCACTCCTCAATTGGATTAGTCTCATCCTTCAATGCTATCATTTCCTTTGATATTGGATCATACTAAGAAACCATTATTATCATGACATTAACCTATAAAAATAGGCGTATCACGAGGCCCTTTCGTC

**Supplementary Figure S1 Full plasmid sequences of the Cas9, gRNA, and**

**donor vectors.** Green, orange, blue, red, light blue, gray letters indicate the promoter, coding sequence, terminator, gRNA, homologous region, and bar gene cassette, respectively. 20-bp target sequences in gRNA are underlined.
