# Supplementary material for: Efficient gene editing in Neurospora crassa with CRISPR technology
Source: Fungal Biol Biotechnol. 2015 Jul 15;2:4. doi: 10.1186/s40694-015-0015-1 (PMC5611662; doi:10.1186/s40694-015-0015-1)
Supplement: Supplementary file 2 — Materials and methods. [file 40694_2015_15_MOESM2_ESM.docx]

***Materials and methods***

**Strains.** Strains used for the experiments are a wild type (74-OR23-1V A: FGSC 2489) from the Fungal Genetics Stock Center (FGSC) and a circadian clock wild type, *ras-1^bd^;a* (328-4), from the laboratory of Dr. J. Dunlap (Dartmouth Medical School, Hanover, NH).

**Materials.** p426-SNR52p-gRNA.CAN1.Y-SUP4t and p415-GalL-Cas9-CYC1t were gifts from Dr. George Church (Addgene plasmid # 43803 and # 43804 ) [1].

**Construction of Cas9, gRNA, and donor plasmid vectors**. gRNA and Cas9 plasmid vectors were modified from p426-SNR52p-gRNA.CAN1.Y-SUP4t and p415-GalL-Cas9-CYC1t, respectively. gRNA, Cas9, and donor vectors were constructed using PCR and yeast recombination. The full-plasmid sequences are shown in Supplementary Figure 1.

**Transformation of *N. crassa*.** A suspension of conidia (2.0 x 10^9^ per ml) was prepared in 1 M sorbitol. A solution containing 5 µg of circular donor plasmids and 0 to 5 µg each of Cas9 and gRNA circular plasmids were mixed with 50 µl of the conidial suspension was transferred to an electroporater cell (BTX Electro Cell Manipulation 600, Genetronics) and shocked by using a charging voltage of 1.5 kV, 600 Ω, and 25 µF. After the electroshock, 0.2 ml of 1 M sorbitol was added, and the solution was spread on agar medium containing Ignite (400 µg/ml). Colonies resistant to Ignite were isolated and tested by PCR using primers to determine whether replacement occurred at the target gene.

**Bioluminescence Assay.** *N. crassa* was grown at 30 °C on plates for 3 to 5 days. In bioluminescence assays, we used standard media containing Vogel’s medium (pH 5.8) [2] with 0.1% glucose, 0.17% arginine, 50 ng/mL biotin, 1.5% (wt/vol) agar, and 12.5 μM luciferin. In vivo luciferase activity was collected for 1 hr exposure with a PIXIS CCD camera from Princeton Instruments controlled by WinView/32 software from Roper Scientific.

**Genomic DNA extraction, PCR, and quantitative PCR.** *N. crassa* was grown in liquid culture media (LCM) containing Vogel’s medium (pH 5.8) with 2% (wt/vol) glucose, 0.5% arginine, and 50 ng/mL biotin for 24 hr, and harvested as previously described [3]. Genomic DNA was isolated with Puregene system (QIAGEN). Purified genomic DNA samples were subjected to PCR or quantitative PCR (qPCR) experiments. qPCR was performed as previously described [3]. The *gh5-1* was used to normalize qPCR data.

**Quantitative RT-PCR.** *N. crassa* was grown in LCM for 24 hr, and harvested as previously described [3]. Total RNA was isolated using Tri Reagent (Molecular Research Center, Inc.), and treated with RQ1 RNase-free DNase (Promega). Quantitative RT-PCR (qRT-PCR) was performed as previously described [3]. The actin mRNA was used to normalize real-time qRT-PCR data.

***References***

1. DiCarlo JE, Norville JE, Mali P, Rios X, Aach J, Church GM. Genome engineering in *Saccharomyces cerevisiae* using CRISPR-Cas systems. *Nucleic Acids Research* 2013;41(7):4336-4343. doi: 10.1093/nar/gkt135.
2. Vogel H. A convenient growth medium for Neurospora (Medium N). Microbial Genet*ics* Bulle*tin* 1956;13: 42-43.
3. Chen CH, Ringelberg CS, Gross RH, Dunlap JC, Loros JJ. Genome‐wide analysis of light‐inducible responses reveals hierarchical light signalling in *Neurospora. EMBO Journal* 2009;28(8):1029-1042. doi: 10.1038/emboj.2009.54.
